# CAGI6 ID panel challenge: assessment of phenotype and variant predictions in 415 children with neurodevelopmental disorders (NDDs)

**DOI:** 10.1007/s00439-024-02722-w

**Published:** 2025-01-09

**Authors:** Maria Cristina Aspromonte, Alessio Del Conte, Shaowen Zhu, Wuwei Tan, Yang Shen, Yexian Zhang, Qi Li, Maggie Haitian Wang, Giulia Babbi, Samuele Bovo, Pier Luigi Martelli, Rita Casadio, Azza Althagafi, Sumyyah Toonsi, Maxat Kulmanov, Robert Hoehndorf, Panagiotis Katsonis, Amanda Williams, Olivier Lichtarge, Su Xian, Wesley Surento, Vikas Pejaver, Sean D. Mooney, Uma Sunderam, Rajgopal Srinivasan, Alessandra Murgia, Damiano Piovesan, Silvio C. E. Tosatto, Emanuela Leonardi

**Affiliations:** 1 Department of Biomedical Sciences, University of Padova, Padova, Italy; 2 Department of Women’s and Children’s Health, University of Padova, Padova, Italy; 3 Department of Electrical and Computer Engineering, Texas A&M University, College Station, TX 77843 USA; 4 CUHK Shenzhen Research Institute, Shenzhen, China; 5 JC School of Public Health and Primary Care, Chinese University of Hong Kong, Hong Kong, SAR China; 6 Biocomputing Group, Department of Pharmacy and Biotechnology, University of Bologna, Bologna, Italy; 7 Department of Agricultural and Food Sciences, University of Bologna, Bologna, Italy; 8 Computational Bioscience Research Center (CBRC), Computer, Electrical and Mathematical Sciences & Engineering Division (CEMSE), King Abdullah University of Science and Technology (KAUST), Thuwal, 23955-6900 Saudi Arabia; 9 Computer Science Department, College of Computers and Information Technology, Taif University, Taif, 26571 Saudi Arabia; 10 Department of Molecular and Human Genetics, Baylor College of Medicine, One Baylor Plaza, Houston, TX 77030 USA; 11 Department of Biomedical Informatics and Medical Education, University of Washington, Seattle, WA 98195 USA; 12 Institute for Genomic Health, Icahn School of Medicine at Mount Sinai, New York, NY 10029 USA; 13 Department of Genetics and Genomic Sciences, Icahn School of Medicine at Mount Sinai, New York, NY 10029 USA; 14 Innovation Labs, Tata Consultancy Services, Hyderabad, India; 15 Institute of Biomembranes, Bioenergetics and Molecular Biotechnologies, National Research Council (CNR- IBIOM), Bari, Italy

## Abstract

The Genetics of Neurodevelopmental Disorders Lab in Padua provided a new intellectual disability (ID) Panel challenge for computational methods to predict patient phenotypes and their causal variants in the context of the Critical Assessment of the Genome Interpretation, 6th edition (CAGI6). Eight research teams submitted a total of 30 models to predict phenotypes based on the sequences of 74 genes (VCF format) in 415 pediatric patients affected by Neurodevelopmental Disorders (NDDs). NDDs are clinically and genetically heterogeneous conditions, with onset in infant age. Here, we assess the ability and accuracy of computational methods to predict comorbid phenotypes based on clinical features described in each patient and their causal variants. We also evaluated predictions for possible genetic causes in patients without a clear genetic diagnosis. Like the previous ID Panel challenge in CAGI5, seven clinical features (ID, ASD, ataxia, epilepsy, microcephaly, macrocephaly, hypotonia), and variants (Pathogenic/Likely Pathogenic, Variants of Uncertain Significance and Risk Factors) were provided. The phenotypic traits and variant data of 150 patients from the CAGI5 ID Panel Challenge were provided as training set for predictors. The CAGI6 challenge confirms CAGI5 results that predicting phenotypes from gene panel data is highly challenging, with AUC values close to random, and no method able to predict relevant variants with both high accuracy and precision. However, a significant improvement is noted for the best method, with recall increasing from 66% to 82%. Several groups also successfully predicted difficult-to-detect variants, emphasizing the importance of variants initially excluded by the Padua NDD Lab.

**Supplementary Information:**

The online version contains supplementary material available at 10.1007/s00439-024-02722-w.

## Introduction

Neurodevelopmental disorders (NDDs) are a class of disorders that affect brain development and function, characterized by significant genetic and clinical variability. Children with NDDs exhibit cognitive, behavioral, and motor developmental delays. NDDs include conditions such as autism spectrum disorder (ASD), intellectual disability (ID), attention deficit hyperactivity disorder, epilepsy, and motor disorders (Morris-Rosendahl and Crocq 2020; Parenti et al. 2020). Multiple NDDs co-occur with brain size abnormalities, such as microcephaly and macrocephaly (Ritchie and Lizarraga 2023). A combination of two or more of these disorders is frequently reported in patients as comorbidities, which share common functional pathways (Parenti et al. 2020).

The etiology of NDDs is associated with various genetic alterations, including chromosomal rearrangements, copy number variations, small insertions or deletions, and point mutations. Currently, the most common molecular diagnostic practice involves the use of different next-generation sequencing (NGS) approaches, such as targeted gene panels, whole exome sequencing (WES), and whole genome sequencing (WGS). Computational approaches have become crucial for the analysis of data generated by these technologies, enabling the prediction of a patient’s phenotype from their genotype and the identification of causal variants against millions of others. However, due to the genetic and clinical complexity of NDDs, a considerable number of children still lack a molecular diagnosis. Deciphering and analyzing the enormous amount of data produced by WES or WGS, including genes not yet associated with the disease, is a challenge (Sun et al. 2015). Cost-effective gene panels have been widely introduced in routine clinical genetic diagnostics where the analysis of genetic data is limited to the selected genes. However, also in this case many patients will have one or more novel variants that have never been detected before. Following the recommendation from the American College of Medical Genetics and Genomics (ACMG), variants can be classified in five standard categories based on criteria using typical types of variant evidence: pathogenic (P), likely pathogenic (LP), uncertain significance (VUS), likely benign (LB), and benign (B) (Richards et al. 2015). However, despite the standardized efforts of the ACMG guidelines for variant interpretation, the classification of novel DNA variants is a difficult and incompletely solved problem posing significant challenges in the practical application of precision medicine (Kim et al. 2019). The Padua Genetics of Neurodevelopmental Disorders Lab at the Department of Woman and Child Health (University Hospital of Padua, Italy) provided a new Intellectual Disability (ID) panel challenge for the sixth edition of the Critical Assessment of Genome Interpretation (CAGI6; URL: https://genomeinterpretation.org/). Similar to the ID Panel challenge in CAGI5, it involved genetic data obtained from a panel of 74 genes applied to a cohort of 150 pediatric patients (Carraro et al. 2019). In CAGI6 the ID panel challenge expanded the cohort to include 415 patients. Predictors had two primary tasks: (a) predict the phenotypes and (b) predict one or more causal variants that explain the patient disease phenotype. The challenge aimed to encourage development of accurate prediction methods for future use in clinical practice, identifying the genetic cause from a phenotype or vice versa in complex and heterogeneous disorders.

The assessment was performed considering the clinical notes for each patient collected by geneticists as well as candidate variants identified by the Padua NDD lab through targeted gene-panel analysis (Aspromonte et al. 2019). Moreover, for cases lacking a clear genetic diagnosis, predicted variants from various groups were evaluated to assess whether some overlooked variants can indeed play a key role in the patient’s phenotype.

## Methods

### Challenge description

The CAGI6 ID panel challenge consists of two tasks, (i) prediction of patient clinical phenotype among seven phenotypic traits, (ii) prediction of one or more causal variants, based on customized gene-panel sequencing of 415 pediatric patients. The 415 VCF files contain exons and flanking intron regions of 74 genes for different patients. Sequence data were produced with the Ion Torrent PGM platform and processed with the Ion Torrent Suite v5.0 software, as described in (Aspromonte et al. 2019). Further information on sequence data processing is available in the VCF files (e.g. genotype quality, coverage or called genotype). The variants have not been filtered, thus VCF files may contain sequencing errors that should be excluded by sequencing or genotype quality parameters. The complete dataset description, selected variants and patient phenotypes are reported in (Aspromonte et al. 2023).

The genetic disorders associated with 74 genes have been grouped into seven phenotypic traits: intellectual disability, autism spectrum disorder, epilepsy, microcephaly, macrocephaly, hypotonia and ataxia. Patient phenotypic traits are based directly on information provided by the patient’s clinician. Each patient can have one or more phenotypic traits. In some cases, the information regarding the phenotypic trait is not available (NA).

Predictors were provided with a tab-delimited text file for submission, in which they would submit a probability for each phenotypic trait (with values ranging from 0, indicating no disease, to 1, indicating disease), as well as the predicted gene panel variant(s). No predictors used the optional standard deviation to indicate confidence for a phenotypic trait prediction. A validation script is also provided for predictors to check correctness of the format before submission.

Data from the previous CAGI5 ID panel challenge, containing sequence information, patient phenotypes and a list of relevant variants from 150 patients were made available to participants for model training. The predictors also have access to the workflow for variants filtering, interpretation and classification (Aspromonte et al. 2019).

### Phenotype prediction assessment

Phenotype prediction is assessed as a binary classification problem for each available phenotype. Predictors were asked to provide a probability for each patient and possible phenotype, with a probability of zero for any missing values. These predictions were compared against the clinical phenotype given in the Padua NDD lab dataset (ground truth), using the following procedure.

To ensure a fair comparison between predictors, the threshold probability for binary classification is selected by maximizing the True Positive Rate (TPR) at a False Positive Rate (FPR) < 10% for each phenotype. This binary classification was then used to compute the Matthews Correlation Coefficient (MCC), precision, recall, and F1 score for each phenotype based on true positives (*tp*), true negatives (*tn*), false positives (*fp*) and false negatives (*fn*) as follows:

$${\text{Precision = }}\frac{{{\text{tp}}}}{{{\text{tp + fp}}}}{\text{ }}$$

$${\text{Recall = }}\frac{{{\text{tp}}}}{{{\text{tp + fn}}}}$$

$${\text{F1}}\,{\text{score = 2}} \cdot \frac{{{\text{Precision}}}}{{{\text{Precision + Recall}}}}\, = \,\frac{{2tp}}{{2tp + fp + tn}}$$

Receiver operating characteristic Area Under the Curve (AUC) values (Bradley 1997) were generated by comparing the experimental ground truth and predicted probability values for each phenotype using 1000 bootstrap iterations to visualize the trade-off between TPR and FPR. This resampling technique allows us to assess the stability and reliability of the performance estimates, especially for unbalanced datasets, and enhances the statistical validity of our results.

The predictors on each phenotype are ranked based on the bootstrapped AUC values and using the average rank.

### Variants prediction assessment

Predictors were also assessed for their ability to establish causal associations between single nucleotide variations (SNVs) and individual patients in the provided VCF files. This is a multi-label classification problem, where each patient can have one or more variants selected by the Padua NDD lab dataset and predictors likewise can identify zero or more variants per patient. Gene panel sequencing on average identified 300 variants per patient in exons and intronic flanking regions. These were filtered based on frequency in the patient cohort and general population and automatically classified into the five ACMG categories (P, LP, VUS, LB, B) using InterVar (Li and Wang 2017). Whenever possible, in particular for VUS variants, additional experiments were performed to update the classification (e.g. segregation analysis, X inactivation pattern, or transcript analysis). For each patient, variants classified as P, LP, or VUS were reported. The patient report also included new or rare variants in genes associated with a high risk of autism and computational evidence supporting their pathogenicity, even if they had been transmitted from healthy parents. These variants have been classified as risk factors (RF), as it is believed that they alone are not capable of causing the disease. Thus, for the CAGI6 ID panel challenge, the NDD Padua Lab provided three subsets of selected variants P/LP, VUS, and RF.

Precision and recall were used for assessment. True positives (*tp*) are cases where the predictor assigned a variant to a patient matching one of the associated variants. False positives (*fp*) occur when the predictor incorrectly assigns a variant to a patient and false negatives (*fn*) represent cases where the predictor failed to identify a variant that should have been associated with a patient. True negatives (*tn*) are not evaluated, as predictors will only output variants that should be associated with the patient. The precision metric is therefore calculated as the ratio of correctly predicted variants over total predicted variants.

### Prediction methods

Eight teams and a total of 30 models submitted predictions for the CAGI6 ID panel challenge. The main software and tools used for variant and phenotype prediction by each submission method are summarized in Table 1 and described below, while the extended technical details are reported in the Supplementary material. Two of the participating teams (SID#6 and SID#7) also participated in the CAGI5 ID panel challenge (Carraro et al. 2019).

**Table 1:** Computational approaches adopted by different groups for the ID panel challenge in CAGI6

Teams		Variants annotation	Filters		Variants effects	Inheritance	Gene-phenotype association	Mathematical model	CAGI5 Training
SID#	Team name		Low quality	Frequency					
1.1	Anonymous	EVIDENCE VEP	N/a	GnomAD < 5%	OMIM, ClinVar, Uniprot. EVIDENCE uses 3Cnet, spliceAI, REVEL	Yes	Patient-gene matrix scores, HPO	PRS	Yes
1.2								Random forest	
1.3								Random forest	
1.4								Scoring method	
1.5								N/a	
1.6								N/a	
2.1	AIBI-CAGI6	N/a	N/a	Yes	DNABERT	N/a	N/a	GCN	Yes
2.2									
2.3									
2.4									
2.5									
2.6									
3.1	BioStat_CUHK	VEP REVEL	N/a	Absent or MAF < 5% in 1000 Genomes; Hom exclusion; 1/415 pz	ClinVar, Phenolyzer, REVEL	N/a	GWAS	PRS	Yes
3.2									
4.1	Bologna Biocomputing Group	VEP, SNPs&GO	N/a	GnomAD < 1%	SIFT, PolyPhen, SNP&GO, LoF variants	N/a	HPO, PhenPath	Correlation	N/a
5.1	Hoehndorf	VEP, CADD	Yes	N/a	ESM, CADD	N/a	Buckets, ESM from training data	ML (ESM)	Yes
5.2									
5.3									
5.4									
5.5									
6.1	Lichtarge	EA	N/a	MAF in gnomAD	Inheritance pattern, EA score, MAF	Yes	ClinVar, HPO, DisGenet, Genecard, Pubmed	Correlation	Yes
6.2					Inheritance pattern, EA score, MAF		N/a	N/a	
6.3					Inheritance pattern, EA score, MAF		ClinVar, HPO, DisGenet, Genecard, Pubmed	Correlation	
6.4					EA, MAF		Training data	PRS	
6.5					EA, MAF		N/a	N/a	
6.6					EA, MAF		Training data	PRS	
7.1	Mooney Radivojac	ANNOVAR	N/a	Filter in < = 1%	LINSIGHT, MutPred2, REVEL	N/a	DISEASES	RFC	Yes
7.2									
8.1	ILHyd	In-house tool VPR	Yes	Yes	In-house tool VPR	Yes	ClinVar, MEDLINE	Correlation	Yes
8.6									

SID: Submission ID; VEP: Ensembl Variant Effect Predictor; CADD: Combined Annotation Dependent Depletion; MAF: Minor Allele Frequency; Hom: homozygous; LoF: Loss of Function; HPO: Human Phenotype Ontology; PRS: polygenic risk score; ESM: Evolutionary Scale Modeling; VPR: Variant Prioritization; EA: Evolutionary Action.ML: Machine Learning; GCN: Graph convolutional network; RFC: Random Forest Classifier. Mathematical models: Naive Bayes, Correlation, Bayesian, Bayesian network, Trait specific; n/a: not available

### Group 1 – 6 models

Group 1 participated with six models. The in-house EVIDENCE (Seo et al. 2020) software and the Ensembl Variant Effect Predictor (VEP) tool (McLaren et al. 2016) was used to annotate, prioritize and analyze more than 100,000 SNVs extracted from the 415 ID panel Variant Call Format (VCF) files. ACMG (Richards et al. 2015) guidelines were considered for classification based on gnomAD minor allele frequency (MAF) in the general population < 5%. Six different predictions were based on Polygenic risk score, Random forest model, prior-probability and optimal threshold pathogenicity score. Variant predictions and enrichment with Human Phenotype Ontology (HPO) (Köhler et al. 2019) terms were used for genotype-phenotype association.

### Group 2 – 6 models

Group 2 started the variant prediction by analyzing the provided BED file along the hg19 genome sequence, with mutated sequences for each patient derived from the VCF files. DNABERT (Ji et al. 2021) was used to obtain sequence-wise gene representations followed by a k-mer tokenization (k = 6). A gene-centric graph representation using a 6-channel gene-gene interaction (GGI) network (Karimi et al. 2020) was used to train a graph convolutional network (GCN) (Kipf and Welling 2017) for message passing and a learnable weighted summation layer for the graph-wise representations. A seven-dimensional sigmoid layer perceptron was used to predict the disease labels, using a random 7:3 training to test set split, with six different configurations submitted. For each version, the top-20 variants based on the attention weights were identified as the candidate variants. Causal variants were predicted using different combinations of frequency-based voting as ensemble models, with the top-6 ensemble models submitted.

### Group 3 − 2 models

Group 3 used VEP (McLaren et al. 2016) and precalculated REVEL scores (Ioannidis et al. 2016) to annotate the raw VCF files, filtering based on: (1) absent or MAF < 5% in 1000 Genomes Project data, (2) not homozygous reference alleles, (3) present in only one sample, (4) protein-altering variants. Pathogenic/likely pathogenic variants from ClinVar (Landrum et al. 2014) as well as top-ranked variants combining the Phenolyzer (Yang et al. 2015) and REVEL scores were identified as putative causative variants. Polygenic risk scores (PRS) were used to predict the seven traits based on previously associated variants from the NHGRI-EBI GWAS Catalog (URL: www.ebi.ac.uk/gwas), IEU Open GWAS platform (URL: gwas.mrcieu.ac.uk), and GWAS Atlas (URL: atlas.ctglab.nl). GWAS summary statistics were used for ASD and epilepsy while Childhood IQ GWAS summary statistics were used for Intellectual disability. Allele effect sizes were also estimated by logistic regression for each phenotype on the training dataset. The two submissions correspond to constructing PRS using existing GWAS summary statistics or using effect size estimates from the training dataset.

### Group 4 − 1 model

The group 4 method consists of three steps: (i) variant annotation, (ii) selection of putative causative variants, (iii) phenotype association. Variant annotation used VEP (McLaren et al. 2016) filtered with SIFT (Ng and Henikoff 2003), PolyPhen (Adzhubei et al. 2013) and gnomAD allele frequency and putative missense variant effects predicted with SNPs&GO (Manfredi et al. 2022). After filtering out variants with MAF > 1%, putative causative variants were selected as: (i) missense variants predicted damaging by SIFT, PolyPhen and SNPs&GO; (ii) Stop-gain variants altering > 75% of the wild-type protein sequence; (iii) frameshift variants. Phenotypes associated with genes containing putative and causative variants were retrieved from HPO (Köhler et al. 2019), manually curated data and PhenPath (Babbi et al. 2019). Six genes were excluded as associated with all seven phenotypes. Gene-phenotype associations were used to predict the phenotypic effect of the putative causative variants, with a binary score for each possible phenotype.

### Group 5 – 5 models

Group 5 submitted five predictions starting by first applying quality control analysis on the variants generated from the sequencing data, filtering out low-depth genotypes. Next, variants were annotated with VEP (McLaren et al. 2016) and precalculated CADD scores. Two different methods were used for the prediction of clinical phenotypes: Buckets and Evolutionary Scale Modeling (ESM). The bucket representation groups variants by genomic locations to regions of predefined size represented as a vector. The ESM protein representation corresponding to a single gene was used for the causal variant prediction. The model was trained using the set of reported variants (P/LP, VUS, or RF) as positive and the rest as negatives.

### Group 6 – 6 models

Group 6 submitted six predictions based on the Evolutionary Action method (Katsonis and Lichtarge 2014) for determining the pathogenic effect of variants, the gene inheritance pattern (dominant *de novo*, autosomal recessive, and X-linked male causality) and the MAF extrapolated from gnomAD and the CAGI5 training data. The genotype-phenotype association was determined using different criteria depending on the model considered. Some models were developed using information on disease inheritance, while others used known gene-trait associations obtained from ClinVar (Landrum et al. 2018), DisGeNet (Piñero et al. 2017), HPO (Köhler et al. 2019), GeneCards (Stelzer et al. 2016), and PubMed data mining.

### Group 7 − 2 models

Group 7 initially annotated variants using ANNOVAR, distinguishing between non-exonic variants, with pathogenicity predicted by LINSIGHT (Huang et al. 2017), and exonic variants, with pathogenicity predicted using MutPred2 (Pejaver et al. 2020) and REVEL (Ioannidis et al. 2016). For each gene in the panel, seven variant-level features were defined as maximum pathogenicity prediction score within each category. The literature-based DISEASES (Pletscher-Frankild et al. 2015) Z-score was used to infer gene-disease associations after min-max normalization (over the full database) to construct a gene-phenotype matrix. The phenotype was predicted only for patients where potentially causative exonic variants had been identified. The CAGI5 ID panel data was used to train phenotype prediction models using the same steps.

### Group 8 − 2 models

Group 8 combined ranking sample variants and phenotype-gene correlations. Variant effects were calculated based on multiple criteria. First, each variant was assigned a gene independent score using VPR (Variant Prioritization), MAF, evolutionary conservation, in silico deleteriousness predictions, and associated disease. For each variant, cutoff scores were calculated from the percentile ranking of similarly scored variants in the ClinVar database and only those above the 60th percentile retained. Literature genotype-phenotype associations were based on PRIORI-T (Rao et al. 2020). For each sample and gene, variants from the filtered subset were ranked by quality, novelty or rarity. Gene scores were computed as the average of two highest scoring variants (recessive model) and highest scoring variant (dominant model). The probability of a phenotype being linked to a sample was based on the ranked gene scores.

## Results

The CAGI6 ID panel challenge was designed to test: (1) the ability of computational methods to predict comorbid phenotypes from targeted gene-panel data, (2) the accuracy of computational methods in predicting causal variants from a set of real genetic data, and (3) the effectiveness of bioinformatics algorithms in improving gene panel data analysis for clinical practice. Additionally, groups may identify variants that were not selected by the Padua NDD Lab, and use them to genetically diagnose the condition of individuals without previous diagnosis.

### Summary of the ID panel dataset

**Fig. 1:**
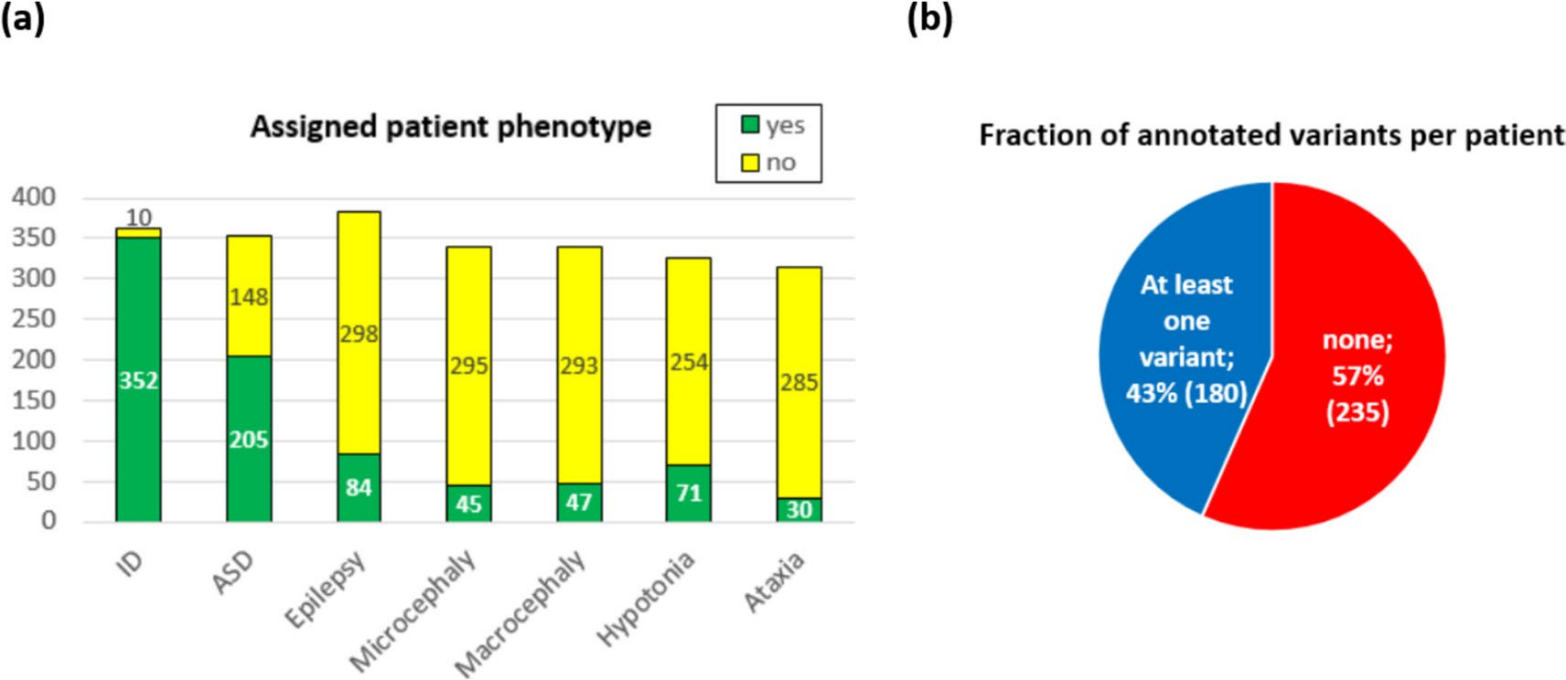
Summary of CAGI-6 ID panel challenge dataset. **a** The number of patients where the presence or absence of the phenotype was ascertained by a clinician. **b** For the 415 patients included in the study, the Padua NDD lab noted at least one variant relevant to the phenotype in 43.4% of the patients

The ID panel dataset includes clinical and genetic data from 415 individuals referred to the Padua NDD Lab. Most patients (84.8%) had intellectual disability (ID), and various phenotypic traits were recorded for different portions of the cohort. Autism spectrum disorder (ASD) was present in 49% of the patients and epilepsy in 20%, making these the next most frequent phenotypes. Some patients exhibited multiple phenotypic traits, with 40% showing both ID and ASD. While clinicians provided information about the presence or absence of these phenotypes for most patients, some data were missing (see Fig. 1).

The Padua NDD Lab identified at least one relevant genetic variant in 180 (43,4%) of the 415 sequenced individuals, resulting in a total of 207 variants (see Fig. 1). These variants were categorized based on their potential effects as follows: pathogenic/likely pathogenic (60 variants), variants of uncertain significance (50 variants), and possible risk factors (97 unique variants). Although pathogenic/likely pathogenic and variants of uncertain significance were unique to individuals, some risk factor variants were found in multiple individuals. Combinations of different variant types within the same patient were rare. All variants were treated equally for the purposes of assessing and ranking predictions.

### Phenotype prediction assessment

#### Overall performance metrics

**Fig. 2:**
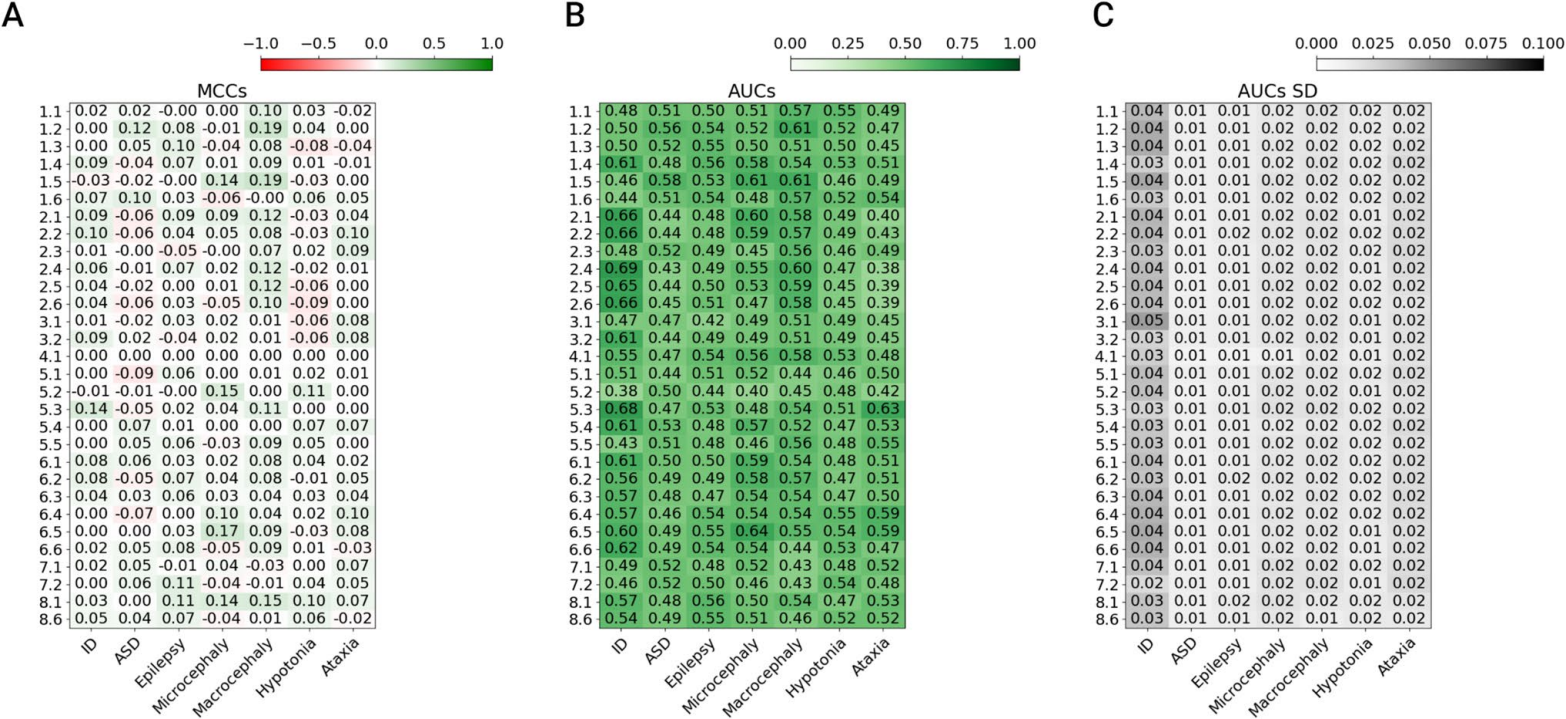
Overall performance for each submission on phenotype prediction. **A** Each cell represents MCC values. The color scale ranges from green (+ 1, perfect correlation) to red (− 1, negative correlation). White means no better than random prediction. **B** Each cell represents the mean AUC values of the ROC for 1000 bootstrap iterations. The color scale ranges from dark (+ 1, perfect performance) to white (0, random performance). **C** Standard deviation (SD) of the bootstrapped AUC values shown in B. AUC, area under ROC curve; MCC, Matthew correlation coefficient; ROC, receiver operating characteristic

**Fig. 3:**
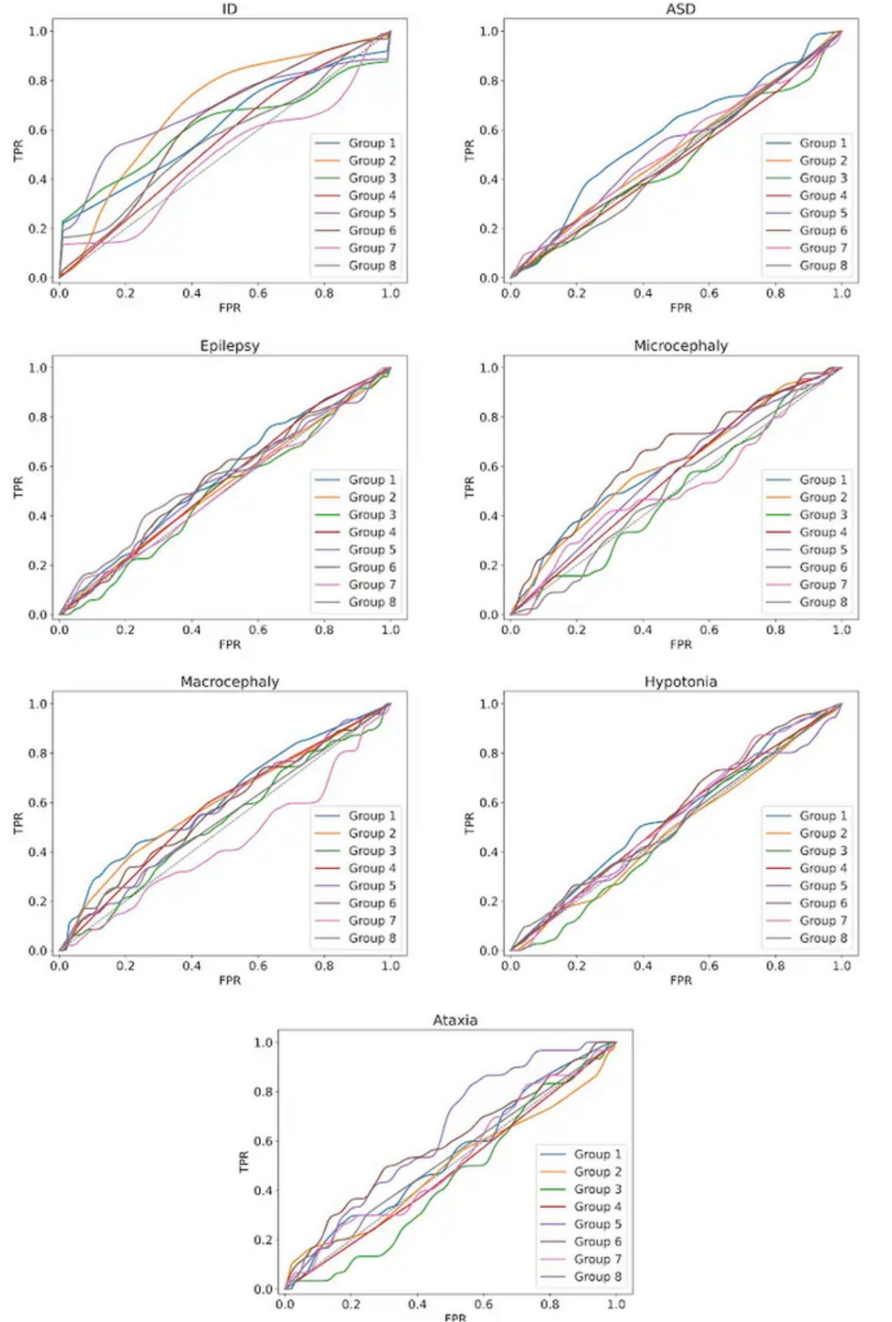
Distribution of the ROC curves for all seven clinical traits. The best performant submission for each phenotype, based on the AUC value, is shown

The first part of the ID-challenge in CAGI6 involved predicting the phenotype for each patient among the seven clinical features. The overall submission performance was assessed using the MCC and AUC values (see Fig. 2) and ROC curve (see Fig. 3) for each phenotype. The AUC standard deviation shows the amount of variation expected in each bootstrap iteration. Precision, recall, and F1 score were also computed for each phenotype (see Supplementary Tables S1-S7). Looking at the results presented in Fig. 2, the overall performance of the predictors is quite underwhelming, with AUC values often very close to a random predictor. There is some prediction signal in the ID phenotype, with a maximum AUC value of 0.69 and a standard deviation of 0.04 for SID#2.4. Although improved performance on the ID phenotype might initially be expected due to the bias in the patient panel, the ROC curve and AUC are not directly influenced by the prevalence of the positive class (ID phenotype). Instead, any observed performance improvement would likely result from the model’s ability to distinguish between ID and non-ID cases, rather than the class distribution. Bootstrapping helps estimate the variance in these metrics but does not necessarily increase the AUC value.

### Performance on different phenotypic traits

**Fig. 4:**
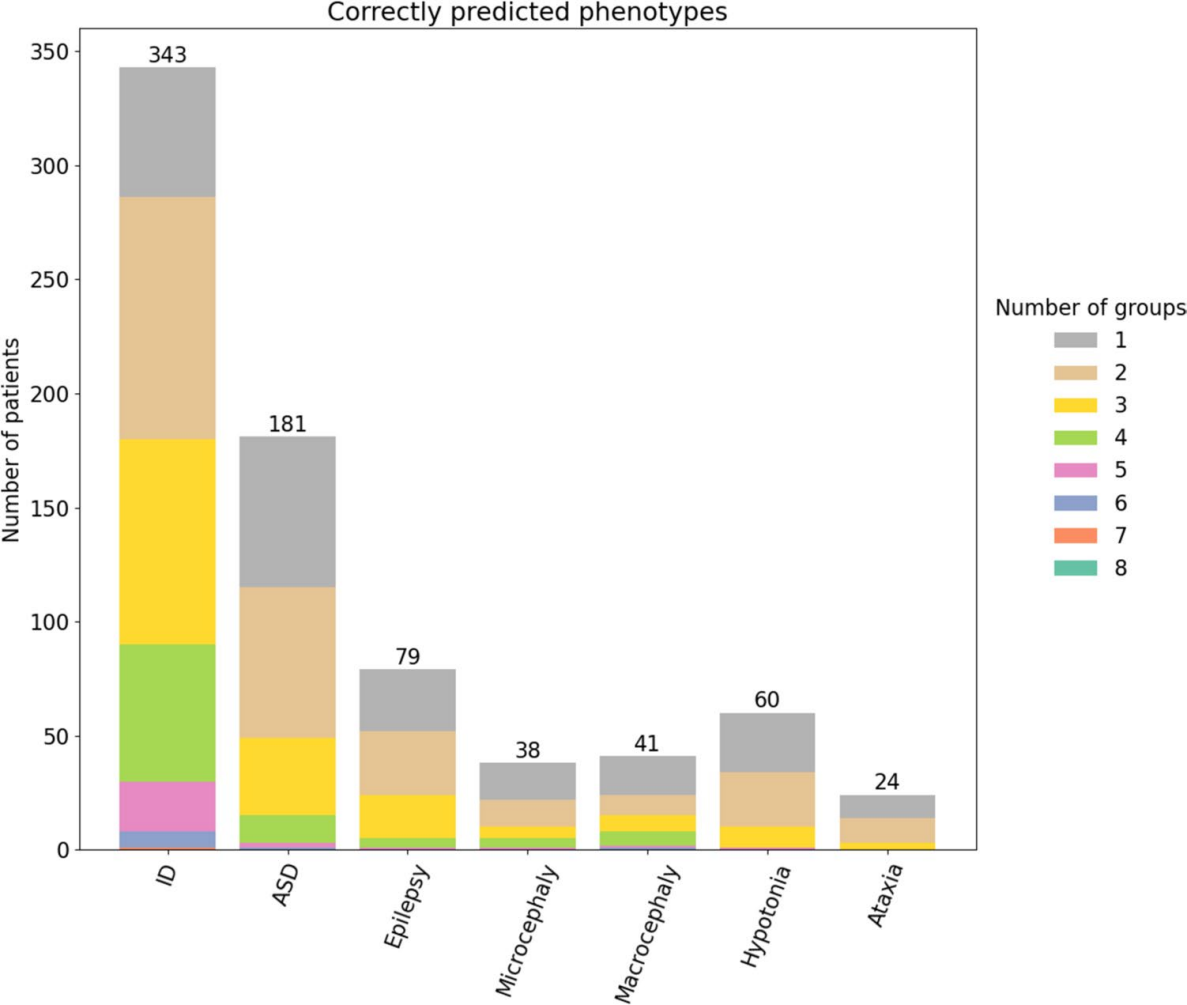
Performance of the eight groups matching the specific phenotype in 415 patients. Colors represent the proportion and number of groups which correctly predicted the phenotype

The phenotype prediction assessment was performed individually for each of the seven traits ascertained by clinicians. Figure 4 shows the number of groups that correctly predicted a patient’s phenotype when it was actually present, using again the threshold for maximum FPR of 10%. Of 352 patients with ID in the cohort, 180 (51%) were correctly identified with the ID phenotype by at least three groups, 110 (30%) by two groups, and 53 (15%) by only one group. The ID phenotype appears to be the easiest to predict, with 343 patients (97%) being predicted by at least one group. In comparison, predictions for other phenotypes range from 80% of patients for Ataxia to 94% for Epilepsy. In most cases, only 30% of patients were correctly detected by a single group.

For different assigned phenotypic traits (see Fig. 2), the ID phenotype was the easiest to match, with SID#2.4 (AUC 0.69) and SID#5.3 (AUC 0.68) performing well. However, even these top predictors had fairly low MCC values of 0.06 and 0.14, respectively, at a maximum FPR of 10%. The second most prevalent trait in our cohort is ASD, reported by clinicians in 205 out of 415 pediatric patients. The highest AUC values for this phenotype were achieved by SID#1.5 (AUC 0.58), SID#1.2 (AUC 0.56) and SID#5.4 (0.53). It is worth noting that the AUC values for ASD remain close to random.

### Comparison of phenotype predictions with CAGI5

The overall submission ranking of this challenge was made considering the average AUC rankings for each phenotype (see Table 2). Comparing the results of MCC and AUC to the previous CAGI5 (Carraro et al. 2019) (see Table S10), we do not notice an improvement in phenotype prediction. However, it should be noted that in CAGI5, no bootstrapping of the ROC curves was performed, and the cutoff threshold was calculated by maximizing the MCC values.

**Table 2:** Ranking of all the predictors based on the ROC AUC values for each phenotype

SID#	ID	ASD	Epilepsy	Microcephaly	Macrocephaly	Hypotonia	Ataxia	Average ranking	Final
1.1	23.02	9.01	17.07	18.87	10.58	3.26	16.28	14.01	12
1.2	21.20	1.94	6.71	16.72	2.35	9.15	20.01	11.15	4
1.3	21.29	6.31	5.80	20.19	21.35	13.69	22.08	15.82	18
1.4	9.03	17.18	3.52	6.53	17.17	5.95	11.23	10.09	2
1.5	24.81	**1.14**	9.56	3.15	**2.20**	23.76	14.70	11.33	5
1.6	26.72	8.86	7.86	24.11	9.66	7.67	6.47	13.05	8
2.1	5.01	27.61	21.68	3.73	7.32	16.97	27.29	15.66	16
2.2	5.40	26.16	23.25	5.66	10.16	16.23	23.84	15.81	17
2.3	22.98	7.03	19.39	27.79	12.74	24.40	14.46	18.40	25
2.4	**3.00**	27.73	20.27	11.07	4.75	22.87	28.55	16.89	20
2.5	6.20	27.47	18.31	14.51	6.37	27.07	28.33	18.32	24
2.6	4.72	24.46	13.89	25.00	6.43	26.98	28.27	18.54	26
3.1	24.46	20.00	29.87	22.20	21.36	17.19	22.24	22.47	29
3.2	9.35	26.97	20.92	22.27	21.22	16.95	22.34	20.00	27
4.1	15.75	19.43	8.43	9.43	7.96	6.03	17.24	12.04	6
5.1	20.15	26.15	16.00	16.37	27.39	25.91	13.65	20.80	28
5.2	29.45	12.51	28.92	29.93	27.04	20.00	26.07	24.85	30
5.3	3.43	19.57	10.44	23.13	15.41	11.48	**1.14**	12.09	7
5.4	9.44	5.12	23.73	6.89	19.59	22.12	8.46	13.62	10
5.5	27.00	8.05	22.38	26.18	11.17	17.73	5.49	16.86	19
6.1	10.10	11.21	19.30	4.79	17.46	18.24	11.52	13.23	9
6.2	15.30	14.90	21.84	6.13	9.28	22.84	11.10	14.49	13
6.3	14.32	17.47	25.65	12.26	16.94	20.67	14.42	17.39	21
6.4	13.96	23.17	6.70	12.15	17.08	**2.39**	2.47	11.13	3
6.5	10.91	14.37	6.10	**1.19**	14.06	5.33	2.90	**7.84**	**1**
6.6	9.04	14.09	7.34	13.05	28.12	6.34	18.93	13.84	11
7.1	22.19	6.42	24.34	16.96	28.41	17.60	9.43	17.91	22
7.2	25.24	7.22	16.66	26.94	28.22	4.28	18.04	18.09	23
8.1	14.33	17.51	**3.42**	20.10	17.46	22.46	7.86	14.73	15
8.6	17.18	15.91	5.66	17.68	25.73	9.44	10.18	14.54	14

Best ranking in each category is shown in bold

For instance, in the case of microcephaly and macrocephaly, where the CAGI6 dataset includes more than twice the number of patients reported with these phenotypes than CAGI5 (see Table 3; Fig. 1), some submissions demonstrated accurate phenotype predictions. SID#6.5 achieved an AUC of 0.64 and a recall of 0.22 for microcephaly (Supplementary Table S5), and SID#1.5 achieved an AUC of 0.61 and a recall of 0.28 for macrocephaly (see Fig. 2 and Supplementary Table S4).

**Table 3:** Patients for whom Padua NDD Lab identified at least one pathogenic/likely pathogenic, VUS, or Risk factor variant in the answer key, summarized by phenotype

Phenotype	Patients	Patients with variants	Pathogenic / Likely pathogenic	Variants of Uncertain Significance	Risk Factor	All unique variants
ID	352	161	58	45	88	182
ASD	205	86	23	21	58	98
Epilepsy	84	36	15	7	21	42
Microcephaly	45	23	9	6	10	24
Macrocephaly	47	18	6	6	9	21
Hypotonia	71	27	11	8	9	28
Ataxia	30	10	5	4	4	13

For each class of variants, the table shows the number of patients who have a variant (Pathogenic / Likely Pathogenic, VUS, or Risk Factor) and the phenotype indicated in the table (e.g., ID, ASD, etc.). There are no unaffected patients in our dataset, but there are patients in whom no variant was found in any of these three classes. Note: Each patient can be associated with more than one phenotype and carry more than one variant

ASD, autism spectrum disorder; ID, intellectual disability; VUS: variant of Uncertain Significance

The CAGI6 cohort reported 71 patients affected by hypotonia, 254 without this phenotype, and 90 for whom information was not available (see Fig. 1). Compared to CAGI5, we did not notice a significant improvement for this phenotype. The maximum AUC across all submissions was 0.55, achieved by SID#1.1 and SID#6.4, attaining a recall of 0.01 and 0.10, respectively (Supplementary Table S6).

The ataxia phenotype was observed in 30 patients, while 285 patients did not exhibit any signs of ataxia (see Fig. 1). The highest-performing model, SID#5.3, has an AUC of 0.63, but fails to attain a maximum FPR of 10% even for the maximum threshold of 1, resulting in a recall and precision equal to 0 (Supplementary Table S7). The AUC results are consistent with the previous assessment. However, it should be mentioned that fewer submissions achieved an AUC score exceeding 0.60 compared to the previous evaluation.

### Phenotype prediction in the subset of patients with identified genetic variants

Similar to CAGI5, we performed an overall phenotype evaluation for patients where the Padua NDD laboratory successfully identified P/LP, VUS, or RF variants. This subset included 180 patients, representing 43.4% of the total cohort (see Fig. 1). The assessment pipeline is the same as before, but now considers only this patient subset. Some changes in prediction performance on the ID phenotype can be appreciated between the entire dataset and this subset, as the percentages of patients predicted by three or more groups raised to 76%, while the overall coverage of predicted patients decreases to 89%. Moreover, we can see an overall improvement regarding the number of groups that correctly identify a patient, with a decrease in the number of patients identified only by one group (see Supplementary Figure S1).

Overall, considering this smaller subset, an improvement of 2.9% was achieved in AUC across the seven phenotypes from all submissions, averaged over all phenotypes. SID#1 achieved the top two positions, while SID#6, which previously held ranks 1 and 3, moved to rank 5 (see Table 2 and Supplementary Table S8).

### Variants prediction assessment

**Fig. 5:**
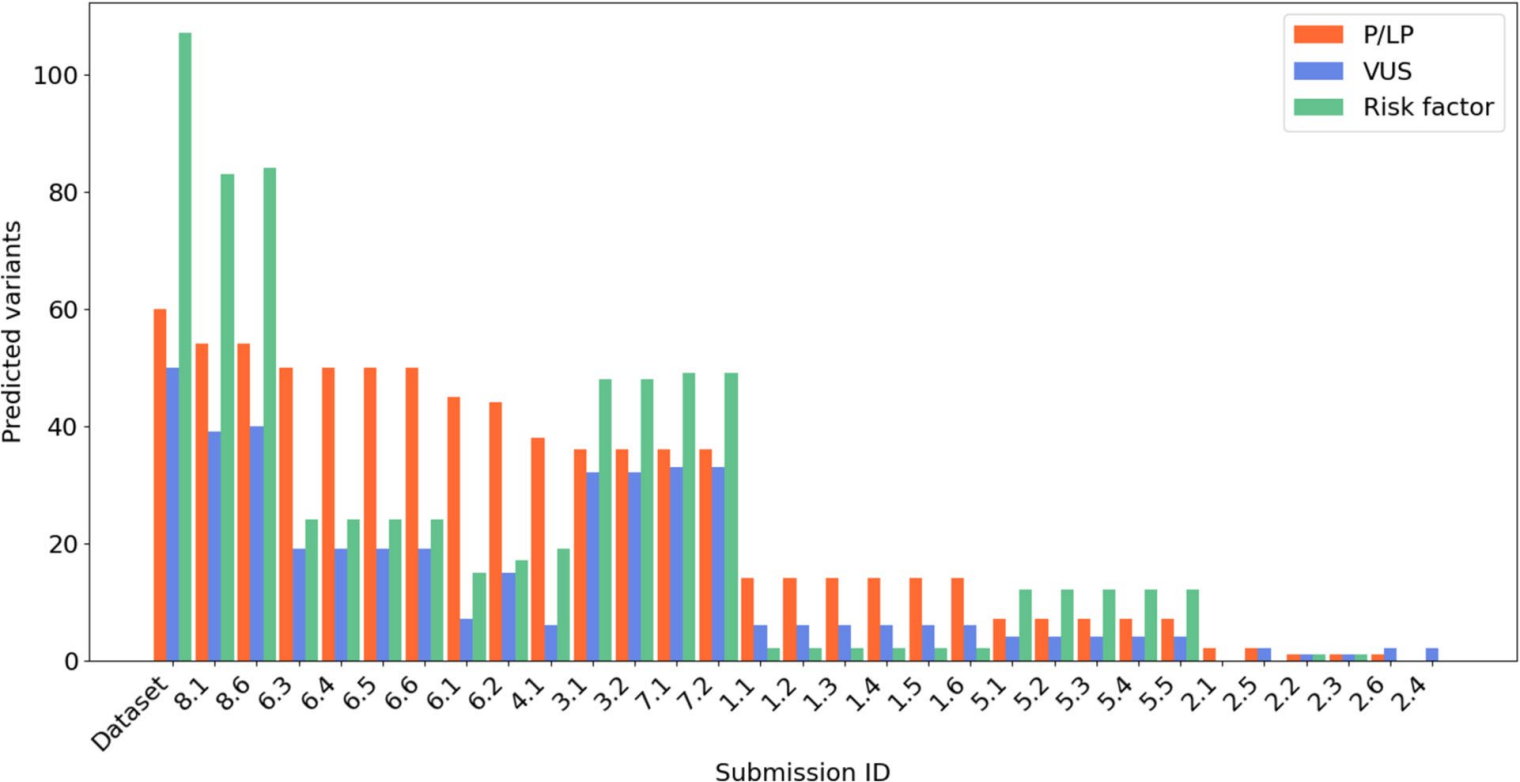
Predicted variants distribution. Category “Dataset” is the amount of variants which were identified and classified by the Padua NDD lab. Each bar represents the amount of variants and types predicted by each submission. NDD, neurodevelopmental disorder

The second part of the CAGI6 challenge was to predict variants associated with the patient phenotype. The overall submission performance was assessed using precision and recall. Figure 5 shows the correctly predicted variants across three classes (P/LP, VUS, RF) for each submission. Groups 8 and 6 correctly predicted most P/LP variants (54 and 52 out of 60, respectively), followed by three other groups (4, 3, 7). Groups 8, 3, and 7 correctly predicted the highest number of VUS and RF variants.

**Fig. 6:**
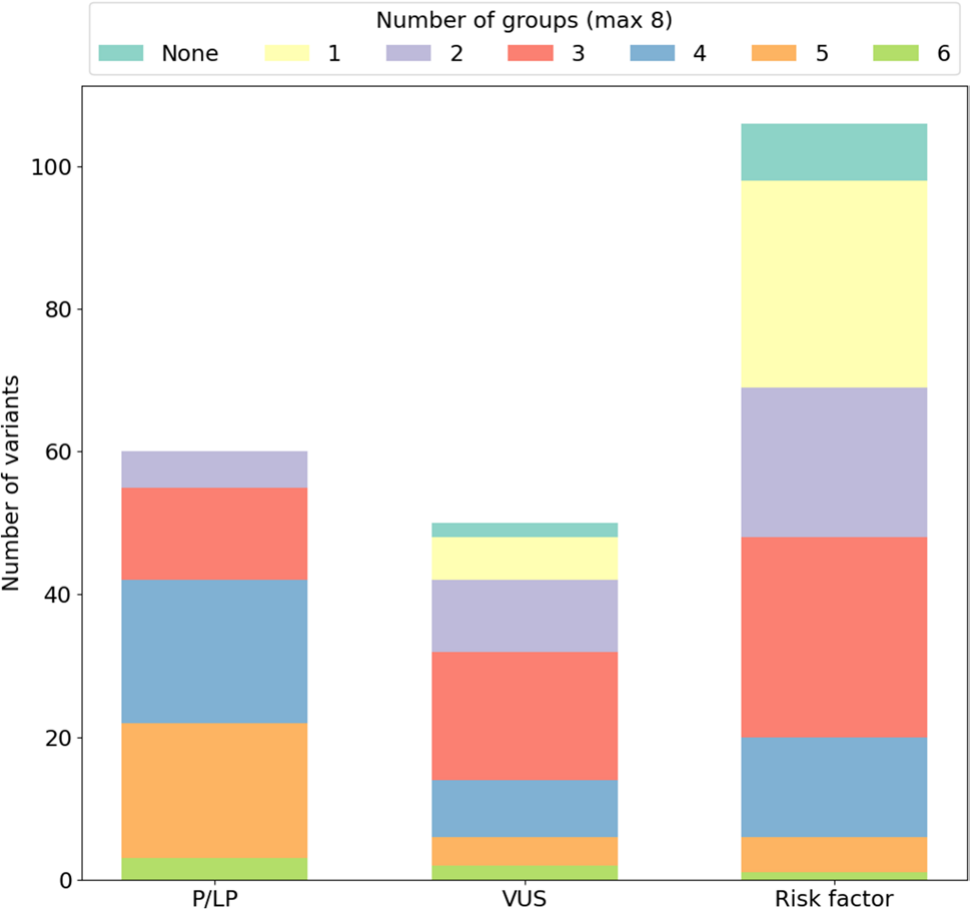
Performance of the eight groups predicting the correct variants. The amount of variants was calculated for each category (P/LP, VUS, RF). Colors indicate the proportion and number of groups which correctly predicted those variants

Figure 6 shows the frequency of each mutation class predicted by different groups. All P/LP variants were predicted by at least two groups (violet), while only 3 were predicted by all groups (green). All VUS were predicted by at least one group, except for the synonymous variant p.Asn839Asn in *CNTNAP2*, which has not been prioritized by any group. Risk factors are overall very sparsely predicted, with 36% predicted only by one group (29 variants) or not predicted at all (8 variants).

Table 4 reports the precision and recall of variant prediction for all submissions. As observed above, SID#8.6 emerges as the most proficient model for capturing a wide range of mutations, exhibiting a recall rate of 82%. However, its precision of 58.7% is lower, implying a significant number of false positives in the results. On the other hand, submission SID#6.2 surpasses all other models with a precision of 72.4%, albeit at a lower recall of 35%, probably due to a limited performance in identifying VUS and RF variants (see Fig. 5). Groups 1, 5, and 2 achieved poor precision and recall in all the three variant classes (see Table 4). This was unexpected, in particular for P/LP variants, considering that the methods developed by group 1 evaluated both the variant frequency and ACMG classification criteria, while group 5 was one of the few to consider filtering variants based on sequencing quality. Additionally, all three groups used the old CAGI5 data to train their methods (see Table 1). Many methods within the same group (e.g. 1, 5, 6) show identical precision and recall as they tend to identify the same variants. This behavior may be due to similarities in the algorithms or criteria used for variant selection.

**Table 4:** Summary of variants prediction assessment by each submission. Highlighted in bold are the best precision and recall values

Submission	Correctly pred. variants	Total pred. variants	Correctly pred. variants/Exp. variants (Recall)	Correctly pred. variants/Total pred. variants (Precision)
1.1	22	627	0.101	0.035
1.2	22	627	0.101	0.035
1.3	22	627	0.101	0.035
1.4	22	627	0.101	0.035
1.5	22	627	0.101	0.035
1.6	22	627	0.101	0.035
2.1	2	181	0.009	0.011
2.2	3	181	0.014	0.017
2.3	3	181	0.014	0.017
2.4	2	181	0.009	0.011
2.5	4	181	0.018	0.022
2.6	3	181	0.014	0.017
3.1	116	232	0.535	0.500
3.2	116	232	0.535	0.500
4.1	63	255	0.290	0.247
5.1	23	181	0.106	0.127
5.2	23	181	0.106	0.127
5.3	23	181	0.106	0.127
5.4	23	181	0.106	0.127
5.5	23	181	0.106	0.127
6.1	67	95	0.309	0.705
6.2	76	105	0.350	**0.724**
6.3	93	131	0.429	0.710
6.4	93	131	0.429	0.710
6.5	93	131	0.429	0.710
6.6	93	131	0.429	0.710
7.1	118	291	0.544	0.405
7.2	118	291	0.544	0.405
8.1	176	341	0.811	0.516
8.6	178	303	**0.820**	0.587

Compared to the CAGI5 challenge, a major improvement can be seen, with the coverage of P/LP variant predictions rising from 64 to 90%, when looking at the respective best model (SID#8.1 for CAGI6 and SID#2.1 for CAGI5). Predictions for VUS and RF variants also improved, rising from 66 to 79% and from 69 to 76%, respectively. Regarding precision for the same models, SID#8.1 achieved 0.516 (Table 4), while in CAGI5 SID#2.1 reached 0.21 (see Table 4 in Carraro et al. 2019).

### Challenges in variant prediction

Multiple groups indicated some variants to predict the phenotypes which we defined as difficult-to-predict (see Supplementary Table S9). These included variants with sequencing parameters indicating possible technical errors, discordant pathogenicity predictions, and deep intronic variants. Initially, variants were filtered based on sequencing parameters and quality (Aspromonte et al. 2019). Two variants were confirmed as pathogenic after Sanger validation and segregation analysis: p.(Arg504Gln) in *GRIN2A*, identified as somatic mosaicism by SID#1, 3, 7, and 8, and p.(Pro1585SerfsTer38) in *SHANK2*, a frameshift deletion initially suspected to be a sequencing error, identified by SID#4 and SID#8.

To prioritize rare missense variants, computational methods were used, including consensus pathogenicity scores from 12 tools and a CADD score (> 25). Although three novel missense variants in *PTCHD1*, *GATAD2B*, and *ASH1L* did not pass this filter, their disease relevance was confirmed through segregation analysis, X-inactivation, and in silico evaluation (Aspromonte et al. 2023). Specifically, for UniPD_0286, the heterozygous *GATAD2B* variant (c.922T > G; p.Cys308Gly) was identified by six groups. The deep intronic variant *MED13L* (c.4956–17 A > G) was correctly predicted by four groups. Transcript analysis showed this variant creates a novel cryptic acceptor site, introducing 16 intronic nucleotides into exon 22 (Aspromonte MC et al. 2023).

### Re-evaluation and classification of predicted variants

One of the objectives for the CAGI6 ID panel challenge was to identify variants that might have been missed by the Padua NDD Lab variant analysis but could still be relevant to the patient phenotypes. The Padua NDD Lab reviewed over 8000 variants, including 3016 exonic, 4520 intronic, 7 splicing, and 137 untranslated region (5′/3′-UTR) variants, linked to at least one patient phenotype. Many variants were excluded due to high prevalence in the cohort or general population (gnomAD) or being classified as sequencing errors (see Supplementary Figure S2). Rare variants were reconsidered for Sanger validation, in silico, or functional analysis.

For the female patient UNIPD_0215, Group 1 and 8 indicated the synonymous variant c.240G > A (p.Leu80Leu) in the *AP1S2* gene. She was suspected of having Smith-Magenis syndrome, presenting developmental delay, ASD, severe intellectual disability, ataxia, dysmorphisms (e.g., synophrys, large mouth), opposite behavior, and poor impulse control. MRI showed a mega cisterna magna and periventricular ischemic dilatation of the ventricular system. These features align with Pettigrew syndrome (MIM# 304340) caused by mutations in the *AP1S2* gene. Human Splicing Finder analysis suggested the variant might alter splicing, leading to its reclassification as likely pathogenic (Aspromonte et al. 2023). However, further segregation and transcript analysis are required to confirm its pathogenicity.

## Discussion

We have reported the assessment of the ID panel challenge for CAGI6 (Critical Assessment of Genome Interpretation Consortium 2024) with the same set-up as in CAGI5 (Aspromonte et al. 2019; Carraro et al. 2019). Sequencing data was provided for 74 genes from which to predict the patient phenotype and infer causal variants. The CAGI6 challenge has a larger cohort (*N* = 415) of pediatric patients with NDDs as well as more participants (8 groups and 30 submissions). Two groups (SID#6 and SID#7) already participated in the CAGI5 ID panel challenge. Predictors were able to train their methods on the CAGI5 ID panel dataset of phenotypes and variants (*N* = 150). The CAGI6 participants used a variety of methods for phenotype predictions, including gene-phenotype association matrices, machine learning approaches, and polygenic risk scores. Variant prediction strategies involved variant annotation, filtering, and functional annotation using genomic data, Gene Ontology terms, or combined tool scores (see Table 1). In assessing phenotype prediction, we used classification thresholds to maximize TPR at FPR < 10%.

ID and ASD were the most common phenotypes, followed by epilepsy, hypotonia, macro/microcephaly and ataxia (see Fig. 1). The prediction of phenotypes, or observable traits, in Neurodevelopmental Disorders (NDDs) is particularly challenging due to the diverse genetic and clinical characteristics exhibited by affected individuals. NDDs encompass a wide range of conditions with varying degrees of severity and symptoms, making it difficult to establish clear patterns between genetic variations and clinical outcomes. Comparatively, predicting phenotypes in NDDs differs significantly from challenges focused on disorders following Mendelian inheritance patterns, such as those encountered in the Hopkins challenge in CAGI4 (Chandonia et al. 2017). Some disorders with clearer genetic associations and inheritance patterns can simplify the prediction process. In our observations, we have noticed that phenotype prediction tends to improve when there is a strong correlation between genotype and disease manifestation. This correlation becomes evident when specific genetic variations consistently lead to particular clinical features or symptoms. In the CAGI6 ID panel challenge, we have demonstrated that the ability to predict the patient phenotype slightly improved in the subset of 180 patients where a P/LP, VUS or RF was identified. While the phenotype can be caused by a primary pathogenic variant, we cannot exclude that other factors (genetic and environmental) may influence the clinical status. In some patients, the phenotype did not fully align with what expected from the alteration of the specific gene, making the diagnosis more difficult. Similar to the CAGI5 dataset, most unexpected findings were related to abnormal head size. This is the case of patients in whom pathogenic variants have been found in the genes *WAC*, *ADNP*, *CHD8* or *MED12* (Aspromonte et al. 2023). There are also cases where dual genetic alterations have complicated the clinical condition, e.g. patient UniPD_0267 had a chromosome alteration (Trisomy X) and a pathogenic variant in *MECP2* (p.Arg270Ter), or case UniPD_0110 with a pathogenic variant in *KDM5C* first diagnosed with fucosidosis syndrome (Leonardi et al. 2023).

Despite the difficulties in predicting phenotypes, some results tend towards an improved prediction power even in rarer phenotypes such as microcephaly and hypotonia. The majority of patients exhibiting these clinical features were predicted by four or more groups (see Fig. 4). This means that more than half of the groups correctly predict these phenotypes.

For the variants assessment we used precision and recall. Accuracy was calculated as the ratio of correctly predicted variants. The prediction of variants had some main protagonists. SID#8 stood out for their predictions of variants, which we have divided into three classes (P/LP, VUS and RF). Other groups also achieved good results (SID#6, SID#4, SID#3, SID#7). Some of them, considered for the filtering and variants prioritization, quality parameters or variants frequency. Many of these groups used data from CAGI5 as training for the new challenge. For example, SID#7, which achieved good predictions for both pathogenic/likely pathogenic and VUS variants, applied a method that involved excluding variants with a frequency greater than 1%. SID#3 also implemented a method that focused on frequency and functional impact of variants (see Fig. 5). On the contrary, SID#1, SID#2, and SID#5 seem to have some difficulties in predicting the three classes of variants. Their methods do not place much importance on variant frequency in the general population or sequencing quality to filter the hundreds of variants present in each VCF. This resulted in the selection of many variants that could be classified as sequencing errors. However, some difficult-to-predict variants were identified. Nonetheless, to infer the phenotype of the genotyped patients, some groups (SID#1, SID#3, SID#6) used a polygenic risk scoring, considering multiple variants from GWAS studies, both rare and common, for specific phenotypic traits. Additionally, we noticed that SID#8, along with SID#1, identified the variant in *AP1S2* as pathogenic. The NDD laboratory in Padua, which initially did not consider this variant, reevaluated it based on splicing prediction and phenotype consistency.

## Conclusions

The current phenotype prediction models exhibit significant limitations, with the best method (SID#2.4) achieving an AUC of 0.69 while many others barely exceed random values. Performance variability across phenotypes suggests a marginal improvement in ID predictions, influenced by a biased dataset. No advancements were observed compared to CAGI5 (Carraro et al. 2019), though a 2.9% improvement in AUC scores was noted when only considering patients with identified variants. Even in variant prediction, despite one group’s strong recall (SID#8.6) and another’s high precision (SID#6.2), achieving both remains challenging. Of note, CAGI6 marked progress with recall increasing to 82% in the best model (SID#8.6). Additionally, models accurately predicted difficult variants, and the re-evaluation of a variant in *AP1S2* by the Padua NDD lab underscored its potential pathogenicity and consistency with patient phenotype. These findings highlight modest improvements and the need for further refinement to enhance prediction accuracy and precision.

## Electronic Supplementary Material

Below is the link to the electronic supplementary material.

Supplementary Material 1.

## References

[CR1] Adzhubei I, Jordan DM, Sunyaev SR (2013) Predicting functional effect of human missense mutations using PolyPhen-2. Curr Protocols Human Genet. 10.1002/0471142905.hg0720s76 10.1002/0471142905.hg0720s76 PMC4480630 23315928

[CR2] Aspromonte MC, Bellini M, Gasparini A, Carraro M, Bettella E, Polli R, Cesca F, Bigoni S, Boni S, Carlet O, Negrin S, Mammi I, Milani D, Peron A, Sartori S, Toldo I, Soli F, Turolla L, Stanzial F, Leonardi E (2019) Characterization of intellectual disability and autism comorbidity through gene panel sequencing. Hum Mutat 40(9):1346–1363. 10.1002/humu.23822 31209962 10.1002/humu.23822 PMC7428836

[CR3] Aspromonte MC, Del Conte A, Polli R, Baldo D, Benedicenti F, Bettella E, Bigoni S, Boni S, Ciaccio C, D’Arrigo S, Donati I (2023) Rare variants in 45 genes account for 25% of cases with NDDs in 415 pediatric patients. 10.21203/rs.3.rs-3139796/v1

[CR4] Babbi G, Martelli PL, Casadio R (2019) PhenPath: a tool for characterizing biological functions underlying different phenotypes. BMC Genomics 20(Suppl 8):548. 10.1186/s12864-019-5868-x 31307376 10.1186/s12864-019-5868-x PMC6631446

[CR5] Bradley AP (1997) The use of the area under the ROC curve in the evaluation of machine learning algorithms. Pattern Recogn 30(7):1145–1159. 10.1016/S0031-3203(96)00142-2

[CR6] Carraro M, Monzon AM, Chiricosta L, Reggiani F, Aspromonte MC, Bellini M, Pagel K, Jiang Y, Radivojac P, Kundu K, Pal LR, Yin Y, Limongelli I, Andreoletti G, Moult J, Wilson SJ, Katsonis P, Lichtarge O, Chen J, Leonardi E (2019) Assessment of patient clinical descriptions and pathogenic variants from gene panel sequences in the CAGI-5 intellectual disability challenge. Hum Mutat 40(9):1330–1345. 10.1002/humu.23823 31144778 10.1002/humu.23823 PMC7341177

[CR7] Chandonia J-M, Adhikari A, Carraro M, Chhibber A, Cutting GR, Fu Y, Gasparini A, Jones DT, Kramer A, Kundu K, Lam HYK, Leonardi E, Moult J, Pal LR, Searls DB, Shah S, Sunyaev S, Tosatto SCE, Yin Y, Buckley BA (2017) Lessons from the CAGI-4 Hopkins clinical panel challenge. Hum Mutat 38(9):1155–1168. 10.1002/humu.23225 28397312 10.1002/humu.23225 PMC5600166

[CR8] Critical Assessment of Genome Interpretation Consortium (2024) CAGI, the critical assessment of genome interpretation, establishes progress and prospects for computational genetic variant interpretation methods. Genome Biol 25:53. 10.1186/s13059-023-03113-6 38389099 10.1186/s13059-023-03113-6 PMC10882881

[CR9] Huang Y-F, Gulko B, Siepel A (2017) Fast, scalable prediction of deleterious noncoding variants from functional and population genomic data. Nat Genet. 10.1038/ng.3810 28288115 10.1038/ng.3810 PMC5395419

[CR10] Ioannidis NM, Rothstein JH, Pejaver V, Middha S, McDonnell SK, Baheti S, Musolf A, Li Q, Holzinger E, Karyadi D, Cannon-Albright LA, Teerlink CC, Stanford JL, Isaacs WB, Xu J, Cooney KA, Lange EM, Schleutker J, Carpten JD, Sieh W (2016) REVEL: an ensemble method for predicting the pathogenicity of rare missense variants. Am J Hum Genet 99(4):877–885. 10.1016/j.ajhg.2016.08.016 27666373 10.1016/j.ajhg.2016.08.016 PMC5065685

[CR11] Ji Y, Zhou Z, Liu H, Davuluri RV (2021) DNABERT: pre-trained bidirectional encoder representations from transformers model for DNA-language in genome. Bioinformatics 37(15):2112–2120. 10.1093/bioinformatics/btab083 33538820 10.1093/bioinformatics/btab083 PMC11025658

[CR12] Katsonis P, Lichtarge O (2014) A formal perturbation equation between genotype and phenotype determines the evolutionary action of protein-coding variations on fitness. Genome Res 24(12):2050–2058. 10.1101/gr.176214.114 25217195 10.1101/gr.176214.114 PMC4248321

[CR13] Kim Y-E, Ki C-S, Jang M-A (2019) Challenges and considerations in sequence variant interpretation for mendelian disorders. Annals Lab Med 39(5):421–429. 10.3343/alm.2019.39.5.421 10.3343/alm.2019.39.5.421 PMC6502951 31037860

[CR14] Kipf TN, Welling M (2017) Semi-supervised classification with graph convolutional networks. arXiv. 10.48550/arXiv.1609.02907

[CR15] Köhler S, Carmody L, Vasilevsky N, Jacobsen JOB, Danis D, Gourdine J-P, Gargano M, Harris NL, Matentzoglu N, McMurry JA, Osumi-Sutherland D, Cipriani V, Balhoff JP, Conlin T, Blau H, Baynam G, Palmer R, Gratian D, Dawkins H, Robinson PN (2019) Expansion of the human phenotype ontology (HPO) knowledge base and resources. Nucleic Acids Res 47(D1):D1018–D1027. 10.1093/nar/gky1105 30476213 10.1093/nar/gky1105 PMC6324074

[CR16] Landrum MJ, Lee JM, Riley GR, Jang W, Rubinstein WS, Church DM, Maglott DR (2014) ClinVar: public archive of relationships among sequence variation and human phenotype. Nucleic Acids Res 42(D1):D980–D985. 10.1093/nar/gkt1113 24234437 10.1093/nar/gkt1113 PMC3965032

[CR17] Landrum MJ, Lee JM, Benson M, Brown GR, Chao C, Chitipiralla S, Gu B, Hart J, Hoffman D, Jang W, Karapetyan K, Katz K, Liu C, Maddipatla Z, Malheiro A, McDaniel K, Ovetsky M, Riley G, Zhou G, Maglott DR (2018) ClinVar: improving access to variant interpretations and supporting evidence. Nucleic Acids Res 46(D1):D1062–D1067. 10.1093/nar/gkx1153 29165669 10.1093/nar/gkx1153 PMC5753237

[CR18] Leonardi E, Aspromonte MC, Drongitis D, Bettella E, Verrillo L, Polli R, McEntagart M, Licchetta L, Dilena R, D’Arrigo S, Ciaccio C, Esposito S, Leuzzi V, Torella A, Baldo D, Lonardo F, Bonato G, Pellegrin S, Stanzial F, Murgia A (2023) Expanding the genetics and phenotypic spectrum of Lysine-specific demethylase 5C (KDM5C): a report of 13 novel variants. Eur J Hum Genet 31(2). 10.1038/s41431-022-01233-4 36434256 10.1038/s41431-022-01233-4 PMC9905063

[CR19] Li Q, Wang K (2017) InterVar: clinical interpretation of genetic variants by the 2015 ACMG-AMP guidelines. Am J Hum Genet 100(2):267–280. 10.1016/j.ajhg.2017.01.004 28132688 10.1016/j.ajhg.2017.01.004 PMC5294755

[CR20] Manfredi M, Savojardo C, Martelli PL, Casadio R (2022) E-SNPs&GO: embedding of protein sequence and function improves the annotation of human pathogenic variants. Bioinformatics 38(23):5168–5174. 10.1093/bioinformatics/btac678 36227117 10.1093/bioinformatics/btac678 PMC9710551

[CR21] McLaren W, Gil L, Hunt SE, Riat HS, Ritchie GRS, Thormann A, Flicek P, Cunningham F (2016) The ensembl variant effect predictor. Genome Biol 17(1):122. 10.1186/s13059-016-0974-4 27268795 10.1186/s13059-016-0974-4 PMC4893825

[CR22] Morris-Rosendahl DJ, Crocq M-A (2020) Neurodevelopmental disorders—the history and future of a diagnostic concept. Dialog Clin Neurosci 22(1):65–72. 10.31887/DCNS.2020.22.1/macrocq 10.31887/DCNS.2020.22.1/macrocq PMC7365295 32699506

[CR23] Ng PC, Henikoff S (2003) SIFT: predicting amino acid changes that affect protein function. Nucleic Acids Res 31(13):3812 12824425 10.1093/nar/gkg509 PMC168916

[CR24] Parenti I, Rabaneda LG, Schoen H, Novarino G (2020) Neurodevelopmental disorders: from genetics to functional pathways. Trends Neurosci. 10.1016/j.tins.2020.05.004 32507511 10.1016/j.tins.2020.05.004

[CR25] Pejaver V, Urresti J, Lugo-Martinez J, Pagel KA, Lin GN, Nam H-J, Mort M, Cooper DN, Sebat J, Iakoucheva LM, Mooney SD, Radivojac P (2020) Inferring the molecular and phenotypic impact of amino acid variants with MutPred2. Nat Commun. 10.1038/s41467-020-19669-x 33219223 10.1038/s41467-020-19669-x PMC7680112

[CR26] Piñero J, Bravo À, Queralt-Rosinach N, Gutiérrez-Sacristán A, Deu-Pons J, Centeno E, García-García J, Sanz F, Furlong LI (2017) DisGeNET: A comprehensive platform integrating information on human disease-associated genes and variants. Nucleic Acids Res 45(Database issue):D833–D839. 10.1093/nar/gkw943 27924018 10.1093/nar/gkw943 PMC5210640

[CR27] Pletscher-Frankild S, Pallejà A, Tsafou K, Binder JX, Jensen LJ (2015) DISEASES: Text mining and data integration of disease–gene associations. Methods 74:83–89. 10.1016/j.ymeth.2014.11.020 25484339 10.1016/j.ymeth.2014.11.020

[CR28] Rao A, Joseph T, Saipradeep VG, Kotte S, Sivadasan N, Srinivasan R (2020) PRIORI-T: A tool for rare disease gene prioritization using MEDLINE. PLoS ONE 15(4):e0231728. 10.1371/journal.pone.0231728 32315351 10.1371/journal.pone.0231728 PMC7173875

[CR29] Richards S, Aziz N, Bale S, Bick D, Das S, Gastier-Foster J, Grody WW, Hegde M, Lyon E, Spector E, Voelkerding K, Rehm HL (2015) Standards and guidelines for the interpretation of sequence variants: a joint consensus recommendation of the american college of medical genetics and genomics and the association for molecular pathology. Genet Sci 17(5):405–424. 10.1038/gim.2015.30 10.1038/gim.2015.30 PMC4544753 25741868

[CR30] Ritchie FD, Lizarraga SB (2023) The role of histone methyltransferases in neurocognitive disorders associated with brain size abnormalities. Front NeuroSci. 10.3389/fnins.2023.989109 36845425 10.3389/fnins.2023.989109 PMC9950662

[CR31] Seo GH, Kim T, Choi IH, Park J, Lee J, Kim S, Won D, Oh A, Lee Y, Choi J, Lee H, Kang HG, Cho HY, Cho MH, Kim YJ, Yoon YH, Eun B-L, Desnick RJ, Keum C, Lee BH (2020) Diagnostic yield and clinical utility of whole exome sequencing using an automated variant prioritization system, EVIDENCE. Clin Genet 98(6):562–570. 10.1111/cge.13848 32901917 10.1111/cge.13848 PMC7756481

[CR32] Stelzer G, Rosen N, Plaschkes I, Zimmerman S, Twik M, Fishilevich S, Stein TI, Nudel R, Lieder I, Mazor Y, Kaplan S, Dahary D, Warshawsky D, Guan-Golan Y, Kohn A, Rappaport N, Safran M, Lancet D (2016) The GeneCards suite: from gene data mining to disease genome sequence analyses. Curr Protocols Bioinf. 10.1002/cpbi.5 10.1002/cpbi.5 27322403

[CR33] Sun Y, Ruivenkamp CAL, Hoffer MJV, Vrijenhoek T, Kriek M, van Asperen CJ, den Dunnen JT, Santen GWE (2015) Next-Generation Diagnostics: Gene Panel, Exome, or Whole Genome? Hum Mutat 36(6):648–655. 10.1002/humu.22783 25772376 10.1002/humu.22783

[CR34] Yang H, Robinson PN, Wang K (2015) Phenolyzer: phenotype-based prioritization of candidate genes for human diseases. Nat Methods 12(9):841–843. 10.1038/nmeth.3484 26192085 10.1038/nmeth.3484 PMC4718403

