# Supplementary material for: CAGI6 ID panel challenge: assessment of phenotype and variant predictions in 415 children with neurodevelopmental disorders (NDDs)
Source: Hum Genet. 2025 Jan 9;144(2-3):227–42. doi: 10.1007/s00439-024-02722-w (PMC11976362; doi:10.1007/s00439-024-02722-w)
Supplement: Supplementary file 1 — Supplementary Material 1. [file 439_2024_2722_MOESM1_ESM.docx]

CAGI6 ID Panel Challenge: Assessment of phenotype and variant predictions in 415 children with Neurodevelopmental Disorders (NDDs)

*Maria Cristina Aspromonte ^1,2^, Alessio Del Conte ^1^, Shaowen Zhu ^3^, Wuwei Tan ^3^, Yang Shen ^3^, Yexian Zhang ^4,5^, Qi Li ^4,5^, Maggie Haitian Wang ^4,5^, Giulia Babbi ^6^, Samuele Bovo ^7^, Pier Luigi Martelli ^6^, Rita Casadio ^6^, Azza Althagafi ^8,9^, Sumyyah Toonsi ^8^, Maxat Kulmanov ^8^, Robert Hoehndorf ^8^, Panagiotis Katsonis ^10^, Amanda Williams ^10^, Olivier Lichtarge ^10^, Su Xian ^11^, Wesley Surento ^11^, Vikas Pejaver ^12^, Sean D. Mooney ^12^, Predrag Radivojac ^12^, Uma Sunderam ^13^ , Rajgopal Srinivasan ^13^, Alessandra Murgia ^2^, Damiano Piovesan ^1^, Silvio C. E. Tosatto ^1,^*, Emanuela Leonardi ^1,2,*^*

***Correspondence: [silvio.tosatto@unipd.it](mailto:silvio.tosatto@unipd.it) (SCET); [emanuela.leonardi@unipd.it](mailto:emanuela.leonardi@unipd.it) (EL)

## SUPPLEMENTARY MATERIAL

## Supplementary Figures


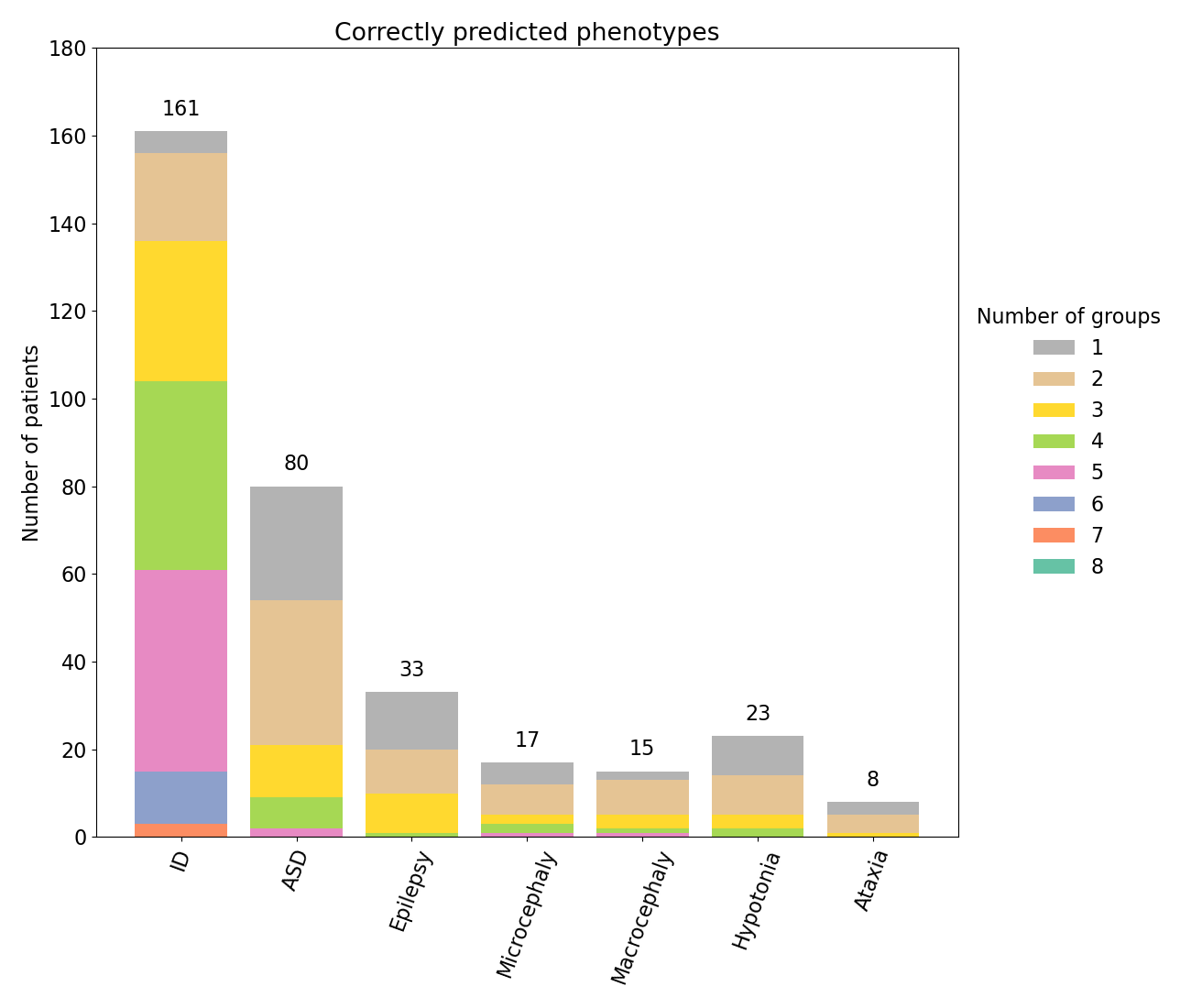


**Supplementary Figure S1 - Performance of the eight groups matching the phenotype in 180 patients carrying only pathogenic/likely pathogenic variants.**  Colours represent the number of different groups correctly predicting the phenotype in patients carrying P/LP variants (legend on the right).


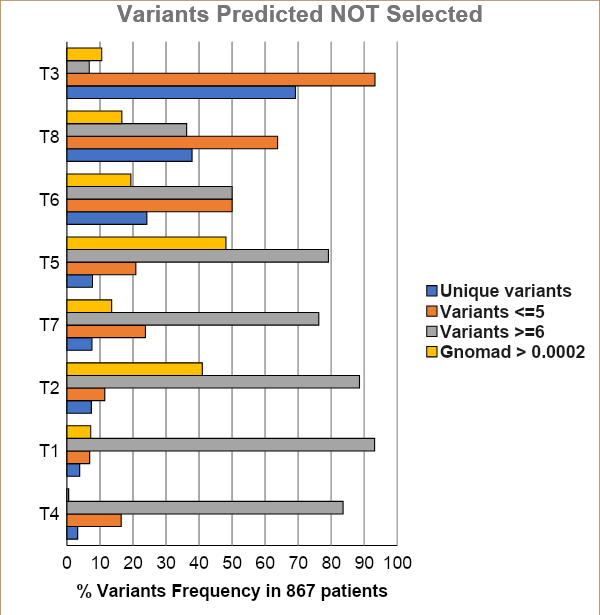


**Supplementary Figure S2 - Variants selected by the predictors to infer phenotypes.** For each team the number of variants with different frequency distributions in the CAGI6 cohort and GnomAD are compared. Groups T3 and T8 reported a high % of unique or rare variants. Other groups (T1 and T4) selected variants that were frequently found in our cohort. As this does not correlate with the frequency of the same variants in the general population, it suggests these variants could be sequencing errors.

## Supplementary Tables

| **SID#** | **MCC** | **AUC** | **Precision** | **Recall** | **F1** |
| --- | --- | --- | --- | --- | --- |
| **1.1** | 0.02 | 0.48 | 0.98 | 0.14 | 0.25 |
| **1.2** | 0.00 | 0.50 | 0.00 | 0.00 | 0.00 |
| **1.3** | 0.00 | 0.50 | 0.00 | 0.00 | 0.00 |
| **1.4** | 0.09 | 0.61 | 1.00 | 0.21 | 0.35 |
| **1.5** | -0.03 | 0.46 | 0.95 | 0.05 | 0.10 |
| **1.6** | 0.07 | 0.44 | 1.00 | 0.15 | 0.26 |
| **2.1** | 0.09 | 0.66 | 0.99 | 0.37 | 0.54 |
| **2.2** | 0.10 | 0.66 | 0.99 | 0.40 | 0.57 |
| **2.3** | 0.01 | 0.48 | 0.98 | 0.12 | 0.22 |
| **2.4** | 0.06 | 0.69 | 0.99 | 0.27 | 0.42 |
| **2.5** | 0.04 | 0.65 | 0.99 | 0.19 | 0.32 |
| **2.6** | 0.04 | 0.66 | 1.00 | 0.05 | 0.10 |
| **3.1** | 0.01 | 0.47 | 1.00 | 0.00 | 0.01 |
| **3.2** | 0.09 | 0.61 | 0.99 | 0.36 | 0.53 |
| **4.1** | 0.00 | 0.55 | 0.00 | 0.00 | 0.00 |
| **5.1** | 0.00 | 0.51 | 0.00 | 0.00 | 0.00 |
| **5.2** | -0.01 | 0.38 | 0.97 | 0.08 | 0.15 |
| **5.3** | 0.14 | 0.68 | 0.99 | 0.53 | 0.69 |
| **5.4** | 0.00 | 0.61 | 0.00 | 0.00 | 0.00 |
| **5.5** | 0.00 | 0.43 | 0.00 | 0.00 | 0.00 |
| **6.1** | 0.08 | 0.61 | 0.99 | 0.31 | 0.48 |
| **6.2** | 0.08 | 0.56 | 0.99 | 0.33 | 0.49 |
| **6.3** | 0.04 | 0.57 | 0.99 | 0.21 | 0.34 |
| **6.4** | 0.00 | 0.57 | 0.00 | 0.00 | 0.00 |
| **6.5** | 0.00 | 0.60 | 0.00 | 0.00 | 0.00 |
| **6.6** | 0.02 | 0.62 | 1.00 | 0.02 | 0.04 |
| **7.1** | 0.02 | 0.49 | 0.98 | 0.14 | 0.25 |
| **7.2** | 0.00 | 0.46 | 0.00 | 0.00 | 0.00 |
| **8.1** | 0.03 | 0.57 | 0.98 | 0.17 | 0.29 |
| **8.6** | 0.05 | 0.54 | 0.99 | 0.24 | 0.38 |
| **Maximum** | **0.14** | **0.69** | **1.00** | **0.53** | **0.69** |

**Supplementary Table S1 - Detailed results for the intellectual disability (ID) phenotype.** The Matthews correlation coefficient (MCC). area under the ROC curve (AUC), precision, recall and F1 values are shown for each submission ID (SID#). The last row shows the maximum value obtained by any group for each parameter.

| **SID#** | **MCC** | **AUC** | **Precision** | **Recall** | **F1** |
| --- | --- | --- | --- | --- | --- |
| **1.1** | 0.02 | 0.51 | 0.62 | 0.09 | 0.15 |
| **1.2** | 0.12 | 0.56 | 0.73 | 0.18 | 0.29 |
| **1.3** | 0.05 | 0.52 | 0.66 | 0.12 | 0.21 |
| **1.4** | -0.04 | 0.48 | 0.50 | 0.05 | 0.09 |
| **1.5** | -0.02 | 0.58 | 0.55 | 0.08 | 0.14 |
| **1.6** | 0.10 | 0.51 | 0.71 | 0.17 | 0.27 |
| **2.1** | -0.06 | 0.44 | 0.44 | 0.03 | 0.06 |
| **2.2** | -0.06 | 0.44 | 0.48 | 0.06 | 0.11 |
| **2.3** | 0.00 | 0.52 | 0.58 | 0.05 | 0.10 |
| **2.4** | -0.01 | 0.43 | 0.56 | 0.02 | 0.05 |
| **2.5** | -0.02 | 0.44 | 0.54 | 0.07 | 0.12 |
| **2.6** | -0.06 | 0.45 | 0.40 | 0.02 | 0.04 |
| **3.1** | -0.02 | 0.47 | 0.55 | 0.08 | 0.14 |
| **3.2** | 0.02 | 0.44 | 0.61 | 0.11 | 0.18 |
| **4.1** | 0.00 | 0.47 | 0.00 | 0.00 | 0.00 |
| **5.1** | -0.09 | 0.44 | 0.42 | 0.05 | 0.09 |
| **5.2** | -0.01 | 0.50 | 0.57 | 0.08 | 0.14 |
| **5.3** | -0.05 | 0.47 | 0.50 | 0.06 | 0.11 |
| **5.4** | 0.07 | 0.53 | 0.67 | 0.14 | 0.23 |
| **5.5** | 0.05 | 0.51 | 0.66 | 0.12 | 0.21 |
| **6.1** | 0.06 | 0.50 | 0.67 | 0.12 | 0.20 |
| **6.2** | -0.05 | 0.49 | 0.50 | 0.07 | 0.12 |
| **6.3** | 0.03 | 0.48 | 0.63 | 0.08 | 0.15 |
| **6.4** | -0.07 | 0.46 | 0.46 | 0.06 | 0.10 |
| **6.5** | 0.00 | 0.49 | 0.59 | 0.10 | 0.17 |
| **6.6** | 0.05 | 0.49 | 0.67 | 0.09 | 0.16 |
| **7.1** | 0.05 | 0.52 | 0.66 | 0.11 | 0.19 |
| **7.2** | 0.06 | 0.52 | 0.66 | 0.13 | 0.22 |
| **8.1** | 0.00 | 0.48 | 0.00 | 0.00 | 0.00 |
| **8.6** | 0.04 | 0.49 | 0.64 | 0.12 | 0.20 |
| **Maximum** | **0.12** | **0.58** | **0.73** | **0.18** | **0.29** |

**Supplementary Table S2 - Detailed results for the Autism spectrum disorder (ASD) phenotype.** The Matthews correlation coefficient (MCC), area under the ROC curve (AUC), precision, recall and F1 values are shown for each submission ID (SID#). The last row shows the maximum value obtained by any group for each parameter.

| **SID#** | **MCC** | **AUC** | **Precision** | **Recall** | **F1** |
| --- | --- | --- | --- | --- | --- |
| **1.1** | 0.00 | 0.50 | 0.22 | 0.10 | 0.13 |
| **1.2** | 0.08 | 0.54 | 0.31 | 0.15 | 0.21 |
| **1.3** | 0.10 | 0.55 | 0.33 | 0.17 | 0.22 |
| **1.4** | 0.07 | 0.56 | 0.33 | 0.10 | 0.15 |
| **1.5** | 0.00 | 0.53 | 0.22 | 0.08 | 0.12 |
| **1.6** | 0.03 | 0.54 | 0.26 | 0.12 | 0.16 |
| **2.1** | 0.09 | 0.48 | 0.36 | 0.10 | 0.15 |
| **2.2** | 0.04 | 0.48 | 0.27 | 0.12 | 0.17 |
| **2.3** | -0.05 | 0.49 | 0.15 | 0.06 | 0.08 |
| **2.4** | 0.07 | 0.49 | 0.35 | 0.07 | 0.12 |
| **2.5** | 0.00 | 0.50 | 0.22 | 0.02 | 0.04 |
| **2.6** | 0.03 | 0.51 | 0.27 | 0.08 | 0.13 |
| **3.1** | 0.03 | 0.42 | 0.33 | 0.02 | 0.04 |
| **3.2** | -0.04 | 0.49 | 0.17 | 0.06 | 0.09 |
| **4.1** | 0.00 | 0.54 | 0.00 | 0.00 | 0.00 |
| **5.1** | 0.06 | 0.51 | 0.29 | 0.14 | 0.19 |
| **5.2** | 0.00 | 0.44 | 0.22 | 0.06 | 0.09 |
| **5.3** | 0.02 | 0.53 | 0.25 | 0.11 | 0.15 |
| **5.4** | 0.01 | 0.48 | 0.23 | 0.10 | 0.13 |
| **5.5** | 0.06 | 0.48 | 0.30 | 0.12 | 0.17 |
| **6.1** | 0.03 | 0.50 | 0.26 | 0.12 | 0.16 |
| **6.2** | 0.07 | 0.49 | 0.32 | 0.12 | 0.17 |
| **6.3** | 0.06 | 0.47 | 0.30 | 0.12 | 0.17 |
| **6.4** | 0.00 | 0.54 | 0.22 | 0.10 | 0.13 |
| **6.5** | 0.03 | 0.55 | 0.27 | 0.08 | 0.13 |
| **6.6** | 0.08 | 0.54 | 0.31 | 0.15 | 0.21 |
| **7.1** | -0.01 | 0.48 | 0.21 | 0.06 | 0.09 |
| **7.2** | 0.11 | 0.50 | 0.38 | 0.14 | 0.21 |
| **8.1** | 0.11 | 0.56 | 0.36 | 0.17 | 0.23 |
| **8.6** | 0.07 | 0.55 | 0.31 | 0.13 | 0.18 |
| **Maximum** | **0.11** | **0.56** | **0.38** | **0.17** | **0.23** |

**Supplementary Table S3 - Detailed results for the Epilepsy phenotype.** The Matthews correlation coefficient (MCC), area under the ROC curve (AUC), precision, recall and F1 values are shown for each submission ID (SID#). The last row shows the maximum value obtained by any group for each parameter.

| **SID#** | **MCC** | **AUC** | **Precision** | **Recall** | **F1** |
| --- | --- | --- | --- | --- | --- |
| **1.1** | 0.10 | 0.57 | 0.25 | 0.17 | 0.20 |
| **1.2** | 0.19 | 0.61 | 0.32 | 0.28 | 0.30 |
| **1.3** | 0.08 | 0.51 | 0.22 | 0.15 | 0.18 |
| **1.4** | 0.09 | 0.54 | 0.23 | 0.15 | 0.18 |
| **1.5** | 0.19 | 0.61 | 0.32 | 0.28 | 0.30 |
| **1.6** | 0.00 | 0.57 | 0.14 | 0.09 | 0.11 |
| **2.1** | 0.12 | 0.58 | 0.26 | 0.19 | 0.22 |
| **2.2** | 0.08 | 0.57 | 0.23 | 0.15 | 0.18 |
| **2.3** | 0.07 | 0.56 | 0.27 | 0.06 | 0.10 |
| **2.4** | 0.12 | 0.60 | 0.26 | 0.19 | 0.22 |
| **2.5** | 0.12 | 0.59 | 0.26 | 0.21 | 0.23 |
| **2.6** | 0.10 | 0.58 | 0.31 | 0.09 | 0.13 |
| **3.1** | 0.01 | 0.51 | 0.15 | 0.09 | 0.11 |
| **3.2** | 0.01 | 0.51 | 0.15 | 0.09 | 0.11 |
| **4.1** | 0.00 | 0.58 | 0.00 | 0.00 | 0.00 |
| **5.1** | 0.01 | 0.44 | 0.15 | 0.11 | 0.12 |
| **5.2** | 0.00 | 0.45 | 0.00 | 0.00 | 0.00 |
| **5.3** | 0.11 | 0.54 | 0.24 | 0.19 | 0.21 |
| **5.4** | 0.00 | 0.52 | 0.00 | 0.00 | 0.00 |
| **5.5** | 0.09 | 0.56 | 0.23 | 0.15 | 0.18 |
| **6.1** | 0.08 | 0.54 | 0.22 | 0.15 | 0.18 |
| **6.2** | 0.08 | 0.57 | 0.22 | 0.15 | 0.18 |
| **6.3** | 0.04 | 0.54 | 0.18 | 0.13 | 0.15 |
| **6.4** | 0.04 | 0.54 | 0.19 | 0.11 | 0.14 |
| **6.5** | 0.09 | 0.55 | 0.33 | 0.06 | 0.11 |
| **6.6** | 0.09 | 0.44 | 0.33 | 0.06 | 0.11 |
| **7.1** | -0.03 | 0.43 | 0.10 | 0.04 | 0.06 |
| **7.2** | -0.01 | 0.43 | 0.13 | 0.09 | 0.10 |
| **8.1** | 0.15 | 0.54 | 0.32 | 0.17 | 0.22 |
| **8.6** | 0.01 | 0.46 | 0.15 | 0.09 | 0.11 |
| **Maximum** | **0.19** | **0.61** | **0.33** | **0.28** | **0.30** |

**Supplementary Table S4 - Detailed results for the Macrocephaly phenotype.** The Matthews correlation coefficient (MCC), area under the ROC curve (AUC), precision, recall and F1 values are shown for each submission ID (SID#). The last row shows the maximum value obtained by any group for each parameter.

| **SID#** | **MCC** | **AUC** | **Precision** | **Recall** | **F1** |
| --- | --- | --- | --- | --- | --- |
| **1.1** | 0.00 | 0.51 | 0.00 | 0.00 | 0.00 |
| **1.2** | -0.01 | 0.52 | 0.12 | 0.07 | 0.09 |
| **1.3** | -0.04 | 0.50 | 0.07 | 0.02 | 0.03 |
| **1.4** | 0.01 | 0.58 | 0.14 | 0.09 | 0.11 |
| **1.5** | 0.14 | 0.61 | 0.27 | 0.22 | 0.24 |
| **1.6** | -0.06 | 0.48 | 0.06 | 0.04 | 0.05 |
| **2.1** | 0.09 | 0.60 | 0.23 | 0.16 | 0.19 |
| **2.2** | 0.05 | 0.59 | 0.19 | 0.13 | 0.16 |
| **2.3** | 0.00 | 0.45 | 0.12 | 0.02 | 0.04 |
| **2.4** | 0.02 | 0.55 | 0.17 | 0.04 | 0.07 |
| **2.5** | 0.01 | 0.53 | 0.14 | 0.04 | 0.07 |
| **2.6** | -0.05 | 0.47 | 0.06 | 0.02 | 0.03 |
| **3.1** | 0.02 | 0.49 | 0.16 | 0.11 | 0.13 |
| **3.2** | 0.02 | 0.49 | 0.16 | 0.11 | 0.13 |
| **4.1** | 0.00 | 0.56 | 0.00 | 0.00 | 0.00 |
| **5.1** | 0.00 | 0.52 | 0.00 | 0.00 | 0.00 |
| **5.2** | 0.15 | 0.40 | 0.50 | 0.07 | 0.12 |
| **5.3** | 0.04 | 0.48 | 0.17 | 0.13 | 0.15 |
| **5.4** | 0.00 | 0.57 | 0.00 | 0.00 | 0.00 |
| **5.5** | -0.03 | 0.46 | 0.10 | 0.07 | 0.08 |
| **6.1** | 0.02 | 0.59 | 0.15 | 0.11 | 0.13 |
| **6.2** | 0.04 | 0.58 | 0.17 | 0.13 | 0.15 |
| **6.3** | 0.03 | 0.54 | 0.17 | 0.09 | 0.12 |
| **6.4** | 0.10 | 0.54 | 0.33 | 0.07 | 0.11 |
| **6.5** | 0.17 | 0.64 | 0.30 | 0.22 | 0.26 |
| **6.6** | -0.05 | 0.54 | 0.06 | 0.02 | 0.03 |
| **7.1** | 0.04 | 0.52 | 0.17 | 0.13 | 0.15 |
| **7.2** | -0.04 | 0.46 | 0.09 | 0.04 | 0.06 |
| **8.1** | 0.14 | 0.50 | 0.29 | 0.18 | 0.22 |
| **8.6** | -0.04 | 0.51 | 0.09 | 0.07 | 0.08 |
| **Maximum** | **0.17** | **0.64** | **0.50** | **0.22** | **0.26** |

**Supplementary Table S5 - Detailed results for the Microcephaly phenotype.** The Matthews correlation coefficient (MCC), area under the ROC curve (AUC), precision, recall and F1 values are shown for each submission ID (SID#). The last row shows the maximum value obtained by any group for each parameter.

| **SID#** | **MCC** | **AUC** | **Precision** | **Recall** | **F1** |
| --- | --- | --- | --- | --- | --- |
| **1.1** | 0.03 | 0.55 | 0.33 | 0.01 | 0.03 |
| **1.2** | 0.04 | 0.52 | 0.27 | 0.13 | 0.17 |
| **1.3** | -0.08 | 0.50 | 0.11 | 0.04 | 0.06 |
| **1.4** | 0.01 | 0.53 | 0.23 | 0.10 | 0.14 |
| **1.5** | -0.03 | 0.46 | 0.18 | 0.07 | 0.10 |
| **1.6** | 0.06 | 0.52 | 0.30 | 0.13 | 0.18 |
| **2.1** | -0.03 | 0.49 | 0.15 | 0.03 | 0.05 |
| **2.2** | -0.03 | 0.49 | 0.17 | 0.06 | 0.09 |
| **2.3** | 0.02 | 0.46 | 0.24 | 0.10 | 0.14 |
| **2.4** | -0.02 | 0.47 | 0.19 | 0.07 | 0.10 |
| **2.5** | -0.06 | 0.45 | 0.08 | 0.01 | 0.02 |
| **2.6** | -0.09 | 0.45 | 0.09 | 0.03 | 0.04 |
| **3.1** | -0.06 | 0.49 | 0.11 | 0.03 | 0.04 |
| **3.2** | -0.06 | 0.49 | 0.11 | 0.03 | 0.04 |
| **4.1** | 0.00 | 0.53 | 0.00 | 0.00 | 0.00 |
| **5.1** | 0.02 | 0.46 | 0.24 | 0.11 | 0.15 |
| **5.2** | 0.11 | 0.48 | 1.00 | 0.01 | 0.03 |
| **5.3** | 0.00 | 0.51 | 0.00 | 0.00 | 0.00 |
| **5.4** | 0.07 | 0.47 | 0.30 | 0.14 | 0.19 |
| **5.5** | 0.05 | 0.48 | 0.29 | 0.11 | 0.16 |
| **6.1** | 0.04 | 0.48 | 0.27 | 0.13 | 0.17 |
| **6.2** | -0.01 | 0.47 | 0.20 | 0.08 | 0.12 |
| **6.3** | 0.03 | 0.47 | 0.26 | 0.11 | 0.16 |
| **6.4** | 0.02 | 0.55 | 0.24 | 0.10 | 0.14 |
| **6.5** | -0.03 | 0.54 | 0.18 | 0.07 | 0.10 |
| **6.6** | 0.01 | 0.53 | 0.23 | 0.10 | 0.14 |
| **7.1** | 0.00 | 0.48 | 0.22 | 0.08 | 0.12 |
| **7.2** | 0.04 | 0.54 | 0.26 | 0.13 | 0.17 |
| **8.1** | 0.10 | 0.47 | 0.40 | 0.08 | 0.14 |
| **8.6** | 0.06 | 0.52 | 0.30 | 0.13 | 0.18 |
| **Maximum** | **0.11** | **0.55** | **1.00** | **0.14** | **0.19** |

**Supplementary Table S6 - Detailed results for the Hypotonia phenotype.** The Matthews correlation coefficient (MCC), area under the ROC curve (AUC), precision, recall and F1 values are shown for each submission ID (SID#). The last row shows the maximum value obtained by any group for each parameter.

| **SID#** | **MCC** | **AUC** | **Precision** | **Recall** | **F1** |
| --- | --- | --- | --- | --- | --- |
| **1.1** | -0.02 | 0.49 | 0.08 | 0.07 | 0.07 |
| **1.2** | 0.00 | 0.47 | 0.00 | 0.00 | 0.00 |
| **1.3** | -0.04 | 0.45 | 0.05 | 0.03 | 0.04 |
| **1.4** | -0.01 | 0.51 | 0.09 | 0.07 | 0.08 |
| **1.5** | 0.00 | 0.49 | 0.00 | 0.00 | 0.00 |
| **1.6** | 0.05 | 0.54 | 0.14 | 0.13 | 0.14 |
| **2.1** | 0.04 | 0.40 | 0.14 | 0.10 | 0.12 |
| **2.2** | 0.10 | 0.43 | 0.29 | 0.07 | 0.11 |
| **2.3** | 0.09 | 0.49 | 0.19 | 0.17 | 0.18 |
| **2.4** | 0.01 | 0.38 | 0.10 | 0.10 | 0.10 |
| **2.5** | 0.00 | 0.39 | 0.00 | 0.00 | 0.00 |
| **2.6** | 0.00 | 0.39 | 0.00 | 0.00 | 0.00 |
| **3.1** | 0.08 | 0.45 | 0.33 | 0.03 | 0.06 |
| **3.2** | 0.08 | 0.45 | 0.33 | 0.03 | 0.06 |
| **4.1** | 0.00 | 0.48 | 0.00 | 0.00 | 0.00 |
| **5.1** | 0.01 | 0.50 | 0.10 | 0.10 | 0.10 |
| **5.2** | 0.00 | 0.42 | 0.00 | 0.00 | 0.00 |
| **5.3** | 0.00 | 0.63 | 0.00 | 0.00 | 0.00 |
| **5.4** | 0.07 | 0.53 | 0.22 | 0.07 | 0.10 |
| **5.5** | 0.00 | 0.55 | 0.00 | 0.00 | 0.00 |
| **6.1** | 0.02 | 0.51 | 0.12 | 0.10 | 0.11 |
| **6.2** | 0.05 | 0.51 | 0.14 | 0.13 | 0.14 |
| **6.3** | 0.04 | 0.50 | 0.15 | 0.07 | 0.09 |
| **6.4** | 0.10 | 0.59 | 0.19 | 0.17 | 0.18 |
| **6.5** | 0.08 | 0.59 | 0.17 | 0.13 | 0.15 |
| **6.6** | -0.03 | 0.47 | 0.06 | 0.03 | 0.04 |
| **7.1** | 0.07 | 0.52 | 0.15 | 0.17 | 0.16 |
| **7.2** | 0.05 | 0.48 | 0.16 | 0.10 | 0.12 |
| **8.1** | 0.07 | 0.53 | 0.16 | 0.17 | 0.16 |
| **8.6** | -0.02 | 0.52 | 0.07 | 0.07 | 0.07 |
| **Maximum** | **0.10** | **0.63** | **0.33** | **0.17** | **0.18** |

**Supplementary Table S7 - Detailed results for the Ataxia phenotype.** The Matthews correlation coefficient (MCC), area under the ROC curve (AUC), precision, recall and F1 values are shown for each submission ID (SID#). The last row shows the maximum value obtained by any group for each parameter.

| **SID#** | **ID** | **ASD** | **Epilepsy** | **Microcephaly** | **Macrocephaly** | **Hypotonia** | **Ataxia** | **Average Ranking** | **Final** |
| --- | --- | --- | --- | --- | --- | --- | --- | --- | --- |
| 1.1 | 9.59 | 11.07 | 22.17 | 14.52 | 15.66 | **1.54** | 14.16 | 12.67 | 7 |
| 1.2 | 27.65 | 5.07 | 11.97 | 5.60 | 2.69 | 3.59 | 18.65 | 10.75 | 3 |
| 1.3 | 27.69 | 5.60 | 11.09 | 7.28 | 14.33 | 4.02 | 11.77 | 11.68 | 6 |
| 1.4 | 13.20 | 4.06 | 13.84 | 8.64 | 28.28 | 2.08 | 24.40 | 13.50 | 12 |
| 1.5 | 29.71 | **1.60** | 8.86 | 1.61 | 2.71 | 10.25 | 9.76 | **9.21** | 1 |
| 1.6 | 21.70 | 7.65 | 3.67 | 13.26 | 5.49 | 12.93 | 2.81 | 9.64 | 2 |
| 2.1 | 3.22 | 11.59 | 23.78 | 17.97 | 20.92 | 18.53 | 29.28 | 17.90 | 22 |
| 2.2 | 4.08 | 15.79 | 23.51 | 17.43 | 24.19 | 15.98 | 29.40 | 18.63 | 24 |
| 2.3 | 24.57 | 20.09 | 18.58 | 21.22 | 12.86 | 26.88 | 25.43 | 21.38 | 27 |
| 2.4 | **1.15** | 19.10 | 19.60 | 23.36 | 13.31 | 18.76 | 26.74 | 17.43 | 20 |
| 2.5 | 4.89 | 22.46 | 20.92 | 26.00 | 17.76 | 23.47 | 27.95 | 20.49 | 26 |
| 2.6 | 5.83 | 28.41 | 7.71 | 27.83 | 6.29 | 11.52 | 16.54 | 14.88 | 16 |
| 3.1 | 16.00 | 28.44 | 29.68 | 26.00 | 10.24 | 22.93 | 23.43 | 22.39 | 29 |
| 3.2 | 14.54 | 25.07 | 28.46 | 25.90 | 10.29 | 22.89 | 23.64 | 21.54 | 28 |
| 4.1 | 26.27 | 16.67 | 5.38 | 7.30 | 18.92 | 6.30 | 8.27 | 12.73 | 8 |
| 5.1 | 16.82 | 20.53 | 11.56 | 22.85 | 23.79 | 22.96 | 15.87 | 19.20 | 25 |
| 5.2 | 28.55 | 22.41 | 24.26 | 29.97 | 25.35 | 25.74 | 22.65 | 25.56 | 30 |
| 5.3 | 5.11 | 14.05 | 13.41 | 19.29 | 4.55 | 20.47 | 2.05 | 11.27 | 4 |
| 5.4 | 17.77 | 4.39 | 26.08 | 3.36 | 10.29 | 26.43 | 18.05 | 15.20 | 18 |
| 5.5 | 24.10 | 15.50 | 26.40 | 22.31 | **1.01** | 7.14 | **1.42** | 13.98 | 15 |
| 6.1 | 10.50 | 3.73 | 11.74 | 6.20 | 19.38 | 14.87 | 13.49 | 11.42 | 5 |
| 6.2 | 15.44 | 25.61 | 14.94 | 12.04 | 14.84 | 12.71 | 10.67 | 15.18 | 17 |
| 6.3 | 7.68 | 13.56 | 17.44 | 11.75 | 7.69 | 22.91 | 9.55 | 12.94 | 9 |
| 6.4 | 13.22 | 14.72 | 17.63 | 17.54 | 20.61 | 7.50 | 16.46 | 15.38 | 19 |
| 6.5 | 21.54 | 12.67 | 5.11 | **1.49** | 21.75 | 18.24 | 16.26 | 13.86 | 14 |
| 6.6 | 4.28 | 22.57 | 3.36 | 7.35 | 26.64 | 12.12 | 20.31 | 13.80 | 13 |
| 7.1 | 20.32 | 10.62 | 27.31 | 18.54 | 20.31 | 26.04 | 6.09 | 18.46 | 23 |
| 7.2 | 21.59 | 7.47 | 12.74 | 28.68 | 23.80 | 19.71 | 8.41 | 17.49 | 21 |
| 8.1 | 14.69 | 28.78 | 2.29 | 8.44 | 18.44 | 15.12 | 6.03 | 13.40 | 11 |
| 8.6 | 13.28 | 25.72 | **1.53** | 11.30 | 22.62 | 11.36 | 5.45 | 13.04 | 10 |

**Supplementary Table S8 - Predictor ranking based on the AUC for each phenotype for the subset of 180 patients with at least one P/LP, VUS or RF variant.** The submission id (SID#) is shown followed by the ranking on each of the seven phenotypes, the average rank across the phenotypes and the corresponding overall ranking. The best value for each category is shown in bold.

| **Variants with bad sequencing parameters** | | | | | | |
| --- | --- | --- | --- | --- | --- | --- |
| **P** | **GENE** | **TYPE** | **CHR** | **Variant** | **SID#** | **SEQ** |
| 0010 | *GRIN2A* | exonic | chr16:9934644:C:T | NM_001134407.3: c.1511G>A: p.Arg504Gln | 1, 3, 7, 8, | GQ:31, DP:310, AF:0.16 |
| 0418 | *SHANK2* | exonic | chr11:70331632:GCACTGCCCGGCGGGGG:G | NM_012309.5: c.4753_4768del: p.Pro1585SerfsTer38 | 4, 8 | GQ:7, DP:11, AF:0.7 |
| **Missense Variants with CADD<25 and Prediction Consensus <6/12 tools** | | | | | | |
| **P** | **GENE** | **TYPE** | **CHR** | **Variant** | **SID#** | **Consensus** |
| 0169 | *PTCHD1* | exonic | chrX:23411707:G:A | NM_173495.3: c.2289C>A: p.Tyr763Ter | 3, 7 | 5/12 |
| 0286 | *GATAD2B* | exonic | chr1:153789043:A:C | NM_020699.4: c.922T>G: p.Cys308Gly | 1, 3, 4, 6, 7, 8 | 3/12 |
| 0337 | *ASH1L* | exonic | chr1:155449482:T:C | NM_018489.3: c.3179A>G: p.Asn1060Ser | 6, 7, 8 | 23.1 |
| **Deep Splicing Variants** | | | | | | |
| **P** | **GENE** | **TYPE** | **CHR** | **Variant** | **SID#** | **HSF** |
| 0383 | *MED13L* | Intronic | chr12:116420425:T:C | NM_015335.4: c.4956-17A>G: p.Ser1652Argfs*1 | 1, 2, 5, 8 | Alters transcript |

**Supplementary Table S9 - Difficult to predict pathogenic/likely pathogenic variants classified by type of difficulty.** The patient ID (P) is shown next to the gene, type of variant (exonic/intronic), chromosome location (chr), variant (reference sequence, nucleotide and amino acid change), submission ID (SID#) and the following additional information. Sequencing parameters (SEQ) include genotype Quality (GQ), DePth of coverage (DP), and Allele Frequency (AF). The consensus column lists the number of agreeing methods (out of 12). HSF is the Human Splicing Finder.

| **SID#** | **ASD** | **Epilepsy** | **Microcephaly** | **Macorcephaly** | **Hypotonia** | **Ataxia** |
| --- | --- | --- | --- | --- | --- | --- |
| **1.1** | 0.57 | 0.51 | 0.53 | 0.57 | 0.49 | 0.46 |
| **2.1** | 0.7 | 0.55 | 0.38 | 0.51 | 0.52 | 0.61 |
| **2.2** | 0.75 | 0.5 | 0.41 | 0.55 | 0.47 | 0.66 |
| **2.3** | 0.71 | 0.56 | 0.39 | 0.49 | 0.51 | 0.66 |
| **2.4** | 0.78 | 0.49 | 0.41 | 0.48 | 0.49 | 0.72 |
| **2.5** | 0.64 | 0.55 | 0.4 | 0.53 | 0.49 | 0.56 |
| **2.6** | 0.74 | 0.46 | 0.41 | 0.49 | 0.5 | 0.66 |
| **3.1** | 0.51 | 0.52 | 0.43 | 0.5 | 0.54 | 0.49 |
| **3.2** | 0.55 | 0.52 | 0.43 | 0.5 | 0.52 | 0.49 |
| **3.3** | 0.68 | 0.48 | 0.44 | 0.5 | 0.52 | 0.49 |
| **4.1** | 0.61 | 0.56 | 0.54 | 0.57 | 0.51 | 0.45 |
| **4.2** | 0.61 | 0.56 | 0.53 | 0.56 | 0.52 | 0.47 |
| **4.3** | 0.68 | 0.56 | 0.56 | 0.63 | 0.56 | 0.46 |
| **5.1** | 0.84 | 0.49 | 0.5 | 0.56 | 0.46 | 0.47 |

**Supplementary Table S10 - CAGI5 ROC AUC results***.* In this previous version of the challenge, the bootstrap of the ROC curves and their corresponding AUC was not implemented into the evaluation pipeline.

## Additional description of prediction methodologies

### Group 1 - 6 models (Anonymous)

Variant annotation, prioritisation, and analysis of over 100,000 SNVs across 417 VCF files were performed using EVIDENCE, an in-house software [(Seo et al., 2020)](https://www.zotero.org/google-docs/?mWQGsp), according to ACMG guidelines. Rare variants (gnomAD frequency <5%) were annotated using VEP [(McLaren et al., 2016)](https://www.zotero.org/google-docs/?mJQpzY) and prioritized based on information such as gene function, inheritance pattern, and relevance of SNVs to disease databases (OMIM, ClinVar, and UniProt). The final classification was based on the ACMG Bayesian score (a float value ranging from 0 to 1). Common SNVs (gnomAD frequency >5%) were filtered out. For phenotype matching, the patient-gene matrix score and HPO terms were used for score prediction.

**Model1. Random forest with polygenic risk scoring concept**

First of all, we used the polygenic risk scoring concept that more than one variant can contribute to a single disease. Variants’ pathogenicity scores under a certain threshold were filtered out. Total summation of variants’ pathogenicity scores was used as training features. And random-forest classifiers were trained by 149 VCF files and predicted 417 VCF files to submit.

**Model2. Random forest model with hyperparameter tuning**

We can modify several options which are called hyperparameters. Random-forest model consists of many decision trees. Changing hyperparameters means changing tree structure such as depth, error rate etc. We modified those hyperparameters and found out the best case that works for the training set. And we used a random-forest model tuned by hyperparameters to predict 417 VCF files.

**Model3. Random forest with canonical transcripts selection**

After annotating VCF files by using EVIDENCE, we selected variants that affect canonical transcripts only. Those canonical transcript variants’ pathogenicity scores were used for training random-forest models and predicted 417 VCF files.

**Model4. Using prior-probability and the odds**

Annotated VCF files have each variants’ pathogenicity score and calculated odds from those pathogenicity scores. From training data, we could get prior probability. Finally, Post probability was calculated using prior-probability and the odds.

**Model5. Simple scoring method based on inhouse database**

We made a pre-mapped gene-phenotype table using our inhouse database, including HPO and OMIM. We considered the relevance between variant and phenotype so that we could give them a weight score. Finally, we prioritized those scores and calculated probability.

**Model6. Optimal threshold pathogenicity score**

Via “Model2”, we found out that cutting off pathogenicity scores under a certain threshold helped the machine learning model to train better. So we continued several tests to find out what is the best threshold through training samples. Based on the test we used those obtained thresholds to filter out pathogenicity scores before training the model. After training the model, 417 VCF files were predicted for 7 phenotypes.

### Group 5 - 5 models (Hoehndorf team)

**5.1 Dataset**

Sequence data for 74 genes from a cohort of 500 patients. The genetic disorders associated with these genes have been grouped into 7 phenotypic traits (intellectual disability, autism spectrum disorder, epilepsy, microcephaly, macrocephaly, hypotonia, ataxia). The pheno-typic traits for each patient are based directly on information provided by the patient’s physician. Each patient can have one or more phenotypic traits.

**5.2 Data preprocessing and annotations**

We performed a quality control (QC) analysis on the variants generated from the gene panel sequencing data with 20 as a threshold. In addition, we filtered out low-depth genotypes (DP *<* 10). After applying these genotype filters, we annotated the variants with VEP [(McLaren et al., 2016)](https://www.zotero.org/google-docs/?YVQv1x), and the precalculated CADD scores [(Kircher et al., 2014)](https://www.zotero.org/google-docs/?PTrhXd). We used CADD for this challenge mainly because CADD showed better performance than most other prediction methods for predicting the variant pathogenicity.

**5.3 Clinical Phenotype prediction**

We made five submissions based on the different gene-phenotype representation and different combinations of features. We mainly utilize two types of features for supervised learning: Buckets and Evolutionary Scale Modeling (ESM) protein embeddings. In the following sections we will introduce each of them separately.

**5.3.1 Buckets**

In this representation scheme, we aim to exploit the approximate location of the variants. We hope that our approximation of the locus of a given variant will help us associate similar variants and identify the site where mutations relate to a phenotype. For this, we use a 1-Dimensional binary vector of size 1834 corresponding to the regions provided in the bed file. When a variant exists in a region, its corresponding position in the vector is set to 1. We cannot afford to have very sparse vectors so we turn to this approximation which proved to provide signal through our experiments with leave-one-out cross-validation. We use a 2-layer neural network for each phenotype separately. We exclude *NA* samples and use grid search with leave-one-out cross-validation to choose the best hyper-parameters. We have several variations to this representation method according to the variants that are included. That is, we either include all the variants that pass our filters or only include non-synonymous variants. We also have a variation where we double the size of the vector to include information about the maximum CADD score (a popular pathogenicity score) corresponding to a region.

**5.3.2 Evolutionary Scale Modeling (ESM) Protein Representations**

We prefer to keep this method as private as possible. Generally speaking, we utilise the ESM (Evolutionary Scale Modeling) [(Rives et al., 2021)](https://www.zotero.org/google-docs/?fQHJxx)embeddings of proteins to train a supervised neural network to predict the phenotypes. Similar to 5.3.1, we exclude *NA* samples and use grid search with leave-one-out cross-validation to choose the best hyper-parameters.

**5.3.3 Models:**

**Model 1**. Buckets of non synonymous variants and corresponding max CADD: In this submission, we use the feature representation explained in 5.3.1 However, we only include non synonymous variants and add the maximum CADD score per region. Hence, we end up with a 1-D vector whose size is double the number of regions in the bed file.

**Model 2**. ESM: In this submission, we use the feature representation explained in 5.3.2 We use a single layer neural network to predict the 7 phenotypes jointly.

**Model 3**. Buckets of non synonymous variants: In this submission, we use the feature representation explained in 5.3.1. However, we only include non synonymous variants.

**Model 4**. Buckets of all variants and corresponding max CADD: In this submission, we use the feature representation explained in 5.3.1. However, we add the maximum CADD score per region. Hence, we end up with a 1-D vector whose size is double the number of regions in the bed file.

**Model 5**. Buckets of all variants: In this submission, we use the feature representation explained in 5.3.1. We include all variants that pass our filters in this representation.

**5.4 Causal variant prediction**

We made one prediction for the causative variants based on the ESM (see section 5.3.2 and 5) representation and different combinations of features. We average the protein representations yielded in 5.3.2 for a given gene to end up with one representation per gene. Then, we include the predicted impact of variants and previously established associated phenotypes reported in ClinVar [(Landrum et al., 2014)](https://www.zotero.org/google-docs/?VaXxio). We use a binary features vector with the classification of variants according to ClinVar (Benign, Likely Benign, Likely Pathogenic, Pathogenic, Conflicting interpretations of pathogenicity, and Uncertain significance). Finally, we use the CADD score as a feature which captures the pathogenicity of the variant. For training the model, we use as a positive set all the variants that are reported as (causative, putative or contributing factor) and the rest are negative set. Since the training data is highly imbalanced, we used 5-fold cross-validation on oversampled data using Synthetic Minority Oversampling Technique (SMOTE) [(Chawla et al., 2002)](https://www.zotero.org/google-docs/?o6Ej3X). SMOTE is an enhanced sampling method that creates synthetic samples based on the nearest neighbours of feature values in the minority class (positive examples in our case). We perform oversampling of the training set in each fold, which prevents the data leakage from the testing set to the training set and reflects how the model trained on a balanced training set would perform when applied to an imbalanced, unseen test set. We performed hyperparameter tuning using grid search, and trained the model with the best parameters using 2 layers neural networks, 5% dropout, and binary cross-entropy loss combined with a *Sigmoid* as a final layer. We evaluated the model using Area under the ROC Curve (AUC), and we achieved 93,22% using the training dataset. The prediction for the causative variants in all the submissions using the same method, and we include the top three predicted variants with the highest prediction scores.

### Group 6 - 6 models (Lichtarge team)

Our team developed six models using two types of automated scoring systems for the needs of this challenge. All models used the Evolutionary Action (EA) method for estimating the pathogenic effect of each variant.

**Models 1, 2, 3**

These models are based on scoring parameters based on disease-inheritance hypotheses, variants frequency and gene-phenotype associations. For the first one they considered:

1. Recessive disease-inheritance, both alleles of the same gene harbours two different causal mutations (compound heterozygous) or the identical causal mutation (homozygous state).
2. Dominant disease-inheritance, one causal mutation (de novo or extremely rare variant) is the monogenic cause for a disease.

Starting from training data (VCF_CAGI5) they set-up scoring parameters: variants with EA<65 were left out from the dominant hypothesis calculations and variants with 65<EA<75 were weighted by 0.5. They estimated the variants pathogenicity according to the Evolutionary Action (EA) method [(Katsonis & Lichtarge, 2014)](https://www.zotero.org/google-docs/?Y5m2KH), and variant frequency using gnomAD data. For the gene-phenotype associations they used ClinVar, DisGeNet, Human Phenotype Ontology (HPO), GeneCards, and literature. There are few differences between the three models used. Model 1 used the training data (VCF_CAGI5) to weight the sources of gene-phenotype association for the voting procedure.

Model 2 used all gene-phenotype associations we gathered following a voting procedure. Model 3 was similar to submission Model 1, without EA variants filter for the dominant hypothesis.

For the final prediction they calculated the probabilities assuming that the frequency of each phenotype in the training dataset was the same. If the calculated score was below a certain cutoff, any variants were provided as prediction.

**Models 4, 5 ,6**

These models are based on polygenic association scores with genetic mutations clustered by gene, Evolutionary Action, and variant allele frequency as used in the training set. The gene-phenotype association was not used. To derive these associations we either excluded the NAs or we included them as meaning "no phenotype". Models 4 and 5 used the overlap of significant associations from these two analyses, in addition Model 5 was trained for male and female patients separately, we predicted male/female status in the training set (as it was not provided) based on the chromosome X variant zygosity of each sample and then applied to the test data. Model 6 used the outcome of the analysis that excluded NA.

### Group 7 - 2 models (Mooney-Radivojac team)

Our approach integrates variant-pathogenicity prediction with gene-disease association prediction to weight variants found in each patient’s gene panel, according to their predicted ability to cause a given phenotype. These prediction scores are then used as features in supervised, phenotype-specific random forest models to make patient-level prediction phenotypes and directly to identify the most likely causal variant.

**Data preprocessing / annotation**

We utilised the CAGI 5 ID panel challenge dataset as a training set. The .vcf files from this dataset and the prediction set (CAGI 6 data set) were annotated with ANNOVAR (Wang *et al*, 2010) on default parameter settings, using the Grch37 assembly reference. ANNOVAR annotations were used to identify the consequences of variants, pathogenicity predictions and the genes that contained them.

**Inferring variant pathogenicity**

We processed the annotated variants using various scoring algorithms designed to predict the variants’ probability for pathogenicity. For variants in non-exonic regions, we assigned LINSIGHT [(Huang et al., 2017)](https://www.zotero.org/google-docs/?vsZKao) scores. For variants in missense variants in exonic regions, we used MutPred2 [(Pejaver et al., 2020)](https://www.zotero.org/google-docs/?0uCO7P) scores for **Submission 1** and REVEL [(Ioannidis et al., 2016)](https://www.zotero.org/google-docs/?9h5IIk) scores for **Submission 2**. For variants in exonic regions, we also applied a threshold to only include variants with an allele frequency of <= 1%, to focus on rare variants [(Carraro et al., 2019)](https://www.zotero.org/google-docs/?6uTb0J). Other classes of variants were excluded.

**Inferring gene-disease associations**

For a given disease phenotype, to assign a score to each gene in the gene panel, we extracted the relevant scores from DISEASES, a database of gene-disease associations, weighted based on their co-mentions in the literature. We first downloaded the full text mining channel file from the DISEASES database (https://diseases.jensenlab.org/Downloads), and for each of the seven phenotypes presented in the challenge, we extracted z-scores for each gene to create gene-phenotype matrix. The z-scores were min-max normalised (over the full DISEASES data set) to range between zero and one.

**Feature representation**

We divided the non-exonic region variants into 6 categories using categories in ANNOVAR’s output files: intronic, ncRNA_intronic, UTR3, ncRNA_exonic, UTR5, splicing. In each of these non-exonic regions, we calculated the maximum variant score (LINSIGHT score) for each of the genes sequenced for the ID panel. These maximum variant scores are then used as features to represent non-exonic regions. For exonic regions, we calculated the maximum variant score (MutPred2 score in Submission 1, REVEL score for Submission 2) for each gene, and used it as a feature for exonic regions.

Thus, seven features are represented for each gene. For instance, gene MTF1 will have these 7 scores: MTF1_intronic, MTF1_ncRNA_intronic, MTF1_UTR3, MTF1_ncRNA_exonic, MTF1_UTR5, MTF1_splicing, and MTF1_exonic. If no variants were found in a given region or if it was unscored, the feature was set to zero. After removing features that were zero across all samples, 150 samples and 221 features remained in the training set (from CAGI5). We then integrated the normalised gene-disease association scores with these features to account for phenotype-specific differences. For each gene, we multiplied the gene-disease association score for the specific disease phenotype with the previously described features (gene_intronic, gene_ncRNA_intronic, gene_UTR3, gene_ncRNA_exonic, gene_UTR5, gene_splicing, gene_exonic).

**Model training**

Individual random forest models were trained for each of the 7 different phenotypes (intellectual disability, autism spectrum disorder, epilepsy, microcephaly, macrocephaly, hypotonia, ataxia), using 80% of the samples from each phenotype for training, and 20% for validation. During the training process, a grid search method was applied to identify optimal parameters for each phenotype.

**Predicting phenotypes and variants at the patient level**

The CAGI 6 data set was pre-processed and transformed into a feature matrix exactly as described above for the training set. For each patient and phenotype, the data was presented to the relevant random forest model and the output scores were assumed to be the probability of phenotype for a given patient. For predictions on disease-causing variants for each phenotype, we focused only on exonic region variants. We selected variants that met a more stringent allele frequency threshold (<= 2e-5). Then we ranked variants based on the

**Model training**

Individual random forest models were trained for each of the 7 different phenotypes (intellectual disability, autism spectrum disorder, epilepsy, microcephaly, macrocephaly, hypotonia, ataxia), using 80% of the samples from each phenotype for training, and 20% for validation. During the training process, a grid search method was applied to identify optimal parameters for each phenotype.

product of the MutPred2 and gene-disease association scores (as described above) and selected the variant with the highest combined score.

**Packages and software environments**

All .vcf files were annotated using ANNOVAR (version date: 2020-06-08). Data visualisation and processes were performed in a python (version 3.8.8) environment involving the pandas module (version 1.2.4). The training of random forest models was conducted with sklearn (version 0.24.1), while miscellaneous statistical calculations and data preprocessing were done using numpy (version 1.19.5).

### Group 8 - 1 model (ILHyd team)

**General Variant Processing**

1. The variants from all training and test cases were combined to generate an unique set of variants
2. Each variant was annotated using an in-house tool “Varant” [(*Varant:An Open Source Tool for Variant Annotation*, https://compbio.berkeley.edu/proj/varant/Home.html)](https://www.zotero.org/google-docs/?9B3ElU).
3. Based on the above annotation each variant was assigned a score in the range [0-1], with 0 being non-deleterious and 1 being deleterious (unpublished method). The method was developed based on variants annotated as Pathogenic/Benign in Clinvar with evidence level 2 or greater. Details will be provided at a later date, if required.
4. Independently variants from the 74 genes in gnomAD and Clinvar (July 2021 release) were collected and steps 2 & 3 above were performed on these variants. For each of the 74 gene variant sets thus scored, percentile cutoffs in the range [1-99] were computed.

5. The score for each of the id-panel variants was transformed to a percentile value using the score distribution computed in 4 above. Note: the transformation was done on a per-gene basis. Thus, for e.g a score of 0.7 could appear in the 50^th^ percentile in one gene while the same score of 0.7 could appear in the 60^th^ percentile in another gene.

6. Only those variants whose scores were in the 60^th^ or above were considered for further analysis

7. A further filtering was done on the variants, wherein only those variants which occurred in no more than 5% of the 567 samples were retained.

**Variant Processing for Individual Samples**

1. The variants in each sample were bucketed into 3 groups

a) HIGH confidence

b) MEDIUM confidence

c) LOW confidence

following an approach presented in [(Damiati et al., 2016)](https://www.zotero.org/google-docs/?J5DoQB). Only variants that were HIGH or MEDIUM confidence were retained for further analysis.

2. The samples were assigned as male or female based on variants in the X chromosome. 3. Some HIGH/MEDIUM confidence variants were found not to be hemizygous in some samples classified as male. These variants were re-classified as LOW confidence

4. A subset of important variants for each sample was generated based on the above criteria and with

score > 60^th^ percentile (refer point 6 in the General Variant Processing section)

**Gene-Phenotype Association**

Based on a literature survey the association of each gene to the 7 phenotypes was reckoned. No attempt was made to weight the association, viz, all associations assume a certainty of 1.

**Identifying Phenotypes and Causal Variants**

1. For each sample we start with the subset of variants the are both high quality and high scoring as described above

2. The variants are ranked by the number of samples they occur in (fewer is better), the variant quality confidence (HIGH > MEDIUM > LOW), the score of the variant (higher is better) 3. The ranked variants are segregated into sets based on the gene in which the variants occur 4. The score for a gene is computed as the average of the two highest scoring variants for genes that follow a recessive inheritance and as the highest scoring variant for genes that follow a dominant inheritance model.

5. The genes are now ranked by their score.

6. The gene score which is in the range [0-1] is interpreted as the probability. 7. The ranked list of genes is iterated over and each of the phenotypes the gene is associated with is assigned as occurring in the sample with probability equal to gene score. Consequently, it is possible that different variants can be assigned to each phenotype, for the same sample. Moreover, in some cases, two or more genes can have the same score and have common phenotype associations. In such cases, 2 or more variants can be assigned to the same phenotype.

8. The SD column is loosely interpreted as confidence and is computed as C * 0.05 + N * 0.1, where C is the variant quality (HIGH=0, MEDIUM=1) and N is the number of samples in which the variant is seen.

In some cases (about 70) no variants satisfying the criteria outlined above were found. Those have been left as * in the submission. In some cases, a single variant with a score > 0.6 was found in a gene that is thought to follow a recessive inheritance model. In such cases, the probability column will have a value < 0.5.

## References

[Carraro, M., Monzon, A. M., Chiricosta, L., Reggiani, F., Aspromonte, M. C., Bellini, M., Pagel, K., Jiang, Y., Radivojac, P., Kundu, K., Pal, L. R., Yin, Y., Limongelli, I., Andreoletti, G., Moult, J., Wilson, S. J., Katsonis, P., Lichtarge, O., Chen, J., … Leonardi, E. (2019). Assessment of patient clinical descriptions and pathogenic variants from gene panel sequences in the CAGI-5 intellectual disability challenge. *Human Mutation*, *40*(9), 1330–1345. https://doi.org/10.1002/humu.23823](https://www.zotero.org/google-docs/?fQbvfq)

[Chawla, N. V., Bowyer, K. W., Hall, L. O., & Kegelmeyer, W. P. (2002). SMOTE: Synthetic Minority Over-sampling Technique. *Journal of Artificial Intelligence Research*, *16*, 321–357. https://doi.org/10.1613/jair.953](https://www.zotero.org/google-docs/?fQbvfq)

[Chen, S., Francioli, L. C., Goodrich, J. K., Collins, R. L., Kanai, M., Wang, Q., Alföldi, J., Watts, N. A., Vittal, C., Gauthier, L. D., Poterba, T., Wilson, M. W., Tarasova, Y., Phu, W., Yohannes, M. T., Koenig, Z., Farjoun, Y., Banks, E., Donnelly, S., … Karczewski, K. J. (2022). *A genome-wide mutational constraint map quantified from variation in 76,156 human genomes* (p. 2022.03.20.485034). bioRxiv. https://doi.org/10.1101/2022.03.20.485034](https://www.zotero.org/google-docs/?fQbvfq)

[Damiati, E., Borsani, G., & Giacopuzzi, E. (2016). Amplicon-based semiconductor sequencing of human exomes: Performance evaluation and optimization strategies. *Human Genetics*, *135*(5), 499–511. https://doi.org/10.1007/s00439-016-1656-8](https://www.zotero.org/google-docs/?fQbvfq)

[Huang, Y.-F., Gulko, B., & Siepel, A. (2017). Fast, scalable prediction of deleterious noncoding variants from functional and population genomic data. *Nat Genet*, *advance online publication*. http://dx.doi.org/10.1038/ng.3810](https://www.zotero.org/google-docs/?fQbvfq)

[Ioannidis, N. M., Rothstein, J. H., Pejaver, V., Middha, S., McDonnell, S. K., Baheti, S., Musolf, A., Li, Q., Holzinger, E., Karyadi, D., Cannon-Albright, L. A., Teerlink, C. C., Stanford, J. L., Isaacs, W. B., Xu, J., Cooney, K. A., Lange, E. M., Schleutker, J., Carpten, J. D., … Sieh, W. (2016). REVEL: An Ensemble Method for Predicting the Pathogenicity of Rare Missense Variants. *American Journal of Human Genetics*, *99*(4), 877–885. https://doi.org/10.1016/j.ajhg.2016.08.016](https://www.zotero.org/google-docs/?fQbvfq)

[Katsonis, P., & Lichtarge, O. (2014). A formal perturbation equation between genotype and phenotype determines the Evolutionary Action of protein-coding variations on fitness. *Genome Research*, *24*(12), 2050–2058. https://doi.org/10.1101/gr.176214.114](https://www.zotero.org/google-docs/?fQbvfq)

[Kircher, M., Witten, D. M., Jain, P., O’Roak, B. J., Cooper, G. M., & Shendure, J. (2014). A general framework for estimating the relative pathogenicity of human genetic variants. *Nature Genetics*, *46*(3), 310–315. https://doi.org/10.1038/ng.2892](https://www.zotero.org/google-docs/?fQbvfq)

[Landrum, M. J., Lee, J. M., Riley, G. R., Jang, W., Rubinstein, W. S., Church, D. M., & Maglott, D. R. (2014). ClinVar: Public archive of relationships among sequence variation and human phenotype. *Nucleic Acids Research*, *42*(D1), D980–D985. https://doi.org/10.1093/nar/gkt1113](https://www.zotero.org/google-docs/?fQbvfq)

[McLaren, W., Gil, L., Hunt, S. E., Riat, H. S., Ritchie, G. R. S., Thormann, A., Flicek, P., & Cunningham, F. (2016). The Ensembl Variant Effect Predictor. *Genome Biology*, *17*(1), 122. https://doi.org/10.1186/s13059-016-0974-4](https://www.zotero.org/google-docs/?fQbvfq)

[Pejaver, V., Urresti, J., Lugo-Martinez, J., Pagel, K. A., Lin, G. N., Nam, H.-J., Mort, M., Cooper, D. N., Sebat, J., Iakoucheva, L. M., Mooney, S. D., & Radivojac, P. (2020). Inferring the molecular and phenotypic impact of amino acid variants with MutPred2. *Nature Communications*, *11*(1), Article 1. https://doi.org/10.1038/s41467-020-19669-x](https://www.zotero.org/google-docs/?fQbvfq)

[Piñero, J., Ramírez-Anguita, J. M., Saüch-Pitarch, J., Ronzano, F., Centeno, E., Sanz, F., & Furlong, L. I. (2020). The DisGeNET knowledge platform for disease genomics: 2019 update. *Nucleic Acids Research*, *48*(D1), D845–D855. https://doi.org/10.1093/nar/gkz1021](https://www.zotero.org/google-docs/?fQbvfq)

[Rives, A., Meier, J., Sercu, T., Goyal, S., Lin, Z., Liu, J., Guo, D., Ott, M., Zitnick, C. L., Ma, J., & Fergus, R. (2021). Biological structure and function emerge from scaling unsupervised learning to 250 million protein sequences. *Proceedings of the National Academy of Sciences*, *118*(15), e2016239118. https://doi.org/10.1073/pnas.2016239118](https://www.zotero.org/google-docs/?fQbvfq)

[Seo, G. H., Kim, T., Choi, I. H., Park, J., Lee, J., Kim, S., Won, D., Oh, A., Lee, Y., Choi, J., Lee, H., Kang, H. G., Cho, H. Y., Cho, M. H., Kim, Y. J., Yoon, Y. H., Eun, B.-L., Desnick, R. J., Keum, C., & Lee, B. H. (2020). Diagnostic yield and clinical utility of whole exome sequencing using an automated variant prioritization system, EVIDENCE. *Clinical Genetics*, *98*(6), 562–570. https://doi.org/10.1111/cge.13848](https://www.zotero.org/google-docs/?fQbvfq)

[Stelzer, G., Rosen, N., Plaschkes, I., Zimmerman, S., Twik, M., Fishilevich, S., Stein, T. I., Nudel, R., Lieder, I., Mazor, Y., Kaplan, S., Dahary, D., Warshawsky, D., Guan-Golan, Y., Kohn, A., Rappaport, N., Safran, M., & Lancet, D. (2016). The GeneCards Suite: From Gene Data Mining to Disease Genome Sequence Analyses. *Current Protocols in Bioinformatics*, *54*(1), 1.30.1-1.30.33. https://doi.org/10.1002/cpbi.5](https://www.zotero.org/google-docs/?fQbvfq)

[*Varant:An Open source tool for variant annotation*. Retrieved 31 May 2024, from https://compbio.berkeley.edu/proj/varant/Home.html](https://www.zotero.org/google-docs/?fQbvfq)
